# A GPCR-neuropeptide axis dampens hyperactive neutrophils by promoting an alternative-like polarization during bacterial infection

**DOI:** 10.1016/j.immuni.2024.01.003

**Published:** 2024-01-30

**Authors:** Naina Gour, Hwan Mee Yong, Aishwarya Magesh, Aishwarya Atakkatan, Felipe Andrade, Stephane Lajoie, Xinzhong Dong

**Affiliations:** 1 Solomon H. Snyder Department of Neuroscience, School of Medicine, Johns Hopkins University, Baltimore, MD; 2 Department of Environmental Health and Engineering, Bloomberg School of Public Health, Johns Hopkins University, Baltimore, MD; 3 Krieger School of Arts and Sciences, Johns Hopkins University, Baltimore, MD; 4 Department of Molecular Microbiology and Immunology, Bloomberg School of Public Health, Johns Hopkins University, Baltimore, MD; 5 Division of Rheumatology, School of Medicine, Johns Hopkins University, Baltimore, MD; 6 Department of Otolaryngology, School of Medicine, Johns Hopkins University, Baltimore, MD; 7 Howard Hughes Medical Institute, Chevy Chase, MD, USA

## Abstract

The notion that neutrophils exist as a homogenous population is being replaced with the knowledge that neutrophils adopt different functional states. Neutrophils can have a pro-inflammatory phenotype or an anti-inflammatory state, but how these states are regulated remains unclear. Here, we demonstrated that the neutrophil-expressed GPCR, Mrgpra1, is a negative regulator of neutrophil bactericidal functions. Mrgpra1-mediated signaling was driven by its ligand, neuropeptide FF (NPFF), which dictated the balance between pro- and anti-inflammatory programming. Specifically, the Mrgpra1-NPFF axis counter-regulated IFNγ-mediated neutrophil polarization during acute lung infection by favoring an alternative-like polarization, suggesting that it may act to balance overzealous neutrophilic responses. Distinct, cross-regulated populations of neutrophils were the primary source of NPFF and IFNγ during infection. As a subset of neutrophils at steady state expressed NPFF, these findings could have broad implications in various infectious and inflammatory diseases. Therefore, a neutrophil-intrinsic pathway determines their cellular fate, function, and magnitude of infection.

## eTOC Blurb

While it is known that pro- and anti-inflammatory neutrophils exist, the mechanisms that dictate their polarization remain unclear. Gour et al. report that neutrophils express a canonical neuronal receptor, Mrgpra1 and a neuropeptide ligand, neuropeptide FF. They find that NPFF-Mrgpra1 signaling inhibits IFNγ-mediated conditioning to promote anti-inflammatory neutrophil polarization, indicating that a neutrophil-intrinsic pathway determines their cellular fate and function.

## Graphical Abstract

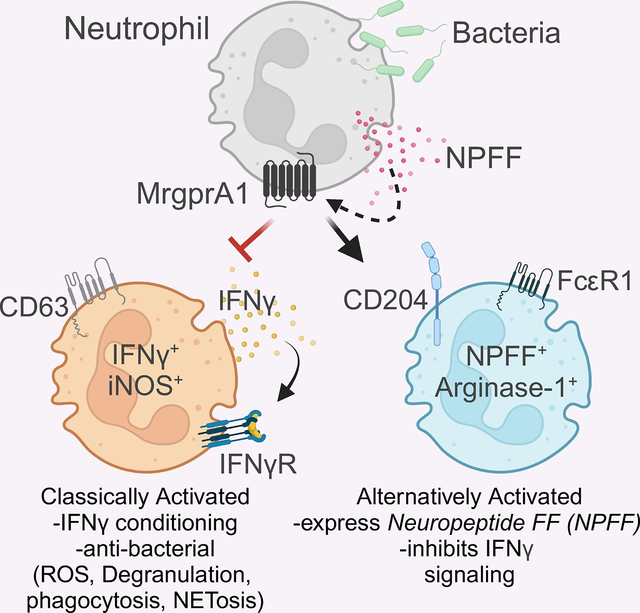

## Introduction

During disease, immune cells can adopt differential polarization states in response to tissue signals that regulate their function. Neutrophils, long thought to be phenotypically homogeneous, can assume polarized states along a spectrum similar to what is found in macrophages^1–3^. Classically activated neutrophils, thought to be primarily induced via IFNγ signaling and TLR stimulation, are enhanced during infection, stroke and myocardial infarction^1, 4–5^. These IFNγ-programmed neutrophils also referred to as N(IFNγ) or N1-like, display a heightened anti-bacterial state characterized by enhanced migration^6^, elevated production of reactive oxygen species (ROS)^6^, and neutrophil extracellular traps (NETs)^6^. Conversely, neutrophils conditioned by type 2 cytokines, sometimes called N2-like, are increased during helminth infection^7^, skin burn^4^ and cancer^2, 3^ where they promote tissue repair and remodeling. Moreover, Th2-conditioned neutrophils have been shown to promote the formation of M2-like macrophages to aid in nematode clearance^7^.

Neutrophil hyperactivation resulting in uncontrolled ROS release and NET formation has been shown to drive manifestations of acute respiratory distress syndrome (ARDS) during bacterial and viral pneumonia. This has been especially prominent in COVID-19-related ARDS, where unchecked, persistent neutrophil activation increased morbidity and mortality^8–13^. We hypothesize that a balanced neutrophil polarization response drives protective immunity during infection while minimizing prolonged inflammation and tissue destruction. However, the mechanisms that regulate divergent functional neutrophil states remain unclear.

The Mas-Related family of G Protein-Coupled Receptors (Mrgprs), including Mrgpra1, are known to be expressed in skin-innervating sensory neurons and regulate itch and pain responses^14,15^. Mrgpra1 has been shown to bind several ligands, including RFamide-neuropeptides, like neuropeptide FF (NPFF), Substance P, somatostatin, and bilirubin^15, 16^. Despite this, the role of Mrgpra1 in immunity remains largely unknown. Conventionally thought to be restricted in expression to neuronal tissue, neuropeptides are now known to be expressed by various non-neuronal cells, such as epithelial cells and immune cells. Regardless of the cellular source, neuropeptide training of immune cells plays a key role in modulating their effector functions at mucosal sites.

Recently Mrgprs were shown to be expressed by immune cells. Notably, the mouse Mrgprb2 is expressed by mast cells^17–21^ where it mediates non-IgE-mediated degranulation^18^, recognizes antimicrobial peptides^20, 21^ and protects against skin bacterial infection^19^. Based on this, we hypothesized that Mrgprs could regulate the function of other innate immune cells. As neutrophils are the most abundant leukocyte and essential for tissue defense, we hypothesized that Mrgprs could regulate the nature and magnitude of activation. Specifically, the biology of Mrgpra1 in immune regulation remains to be studied.

Our findings uncover a key role for Mrgpra1 signaling in neutrophils that controls their activation during bacterial pneumoniae. We find that at both steady state and during infection, distinct populations of neutrophils express NPFF and IFNγ which are associated with divergent polarized states. Importantly, the Mrgpra1-NPFF axis promotes alternative differentiation and regulates the magnitude of IFNγ-induced anti-microbial programming.

## Results

### Neutrophil-expressed *Mrgpra1* prevents their hyperactivation.

**Figure 1.**
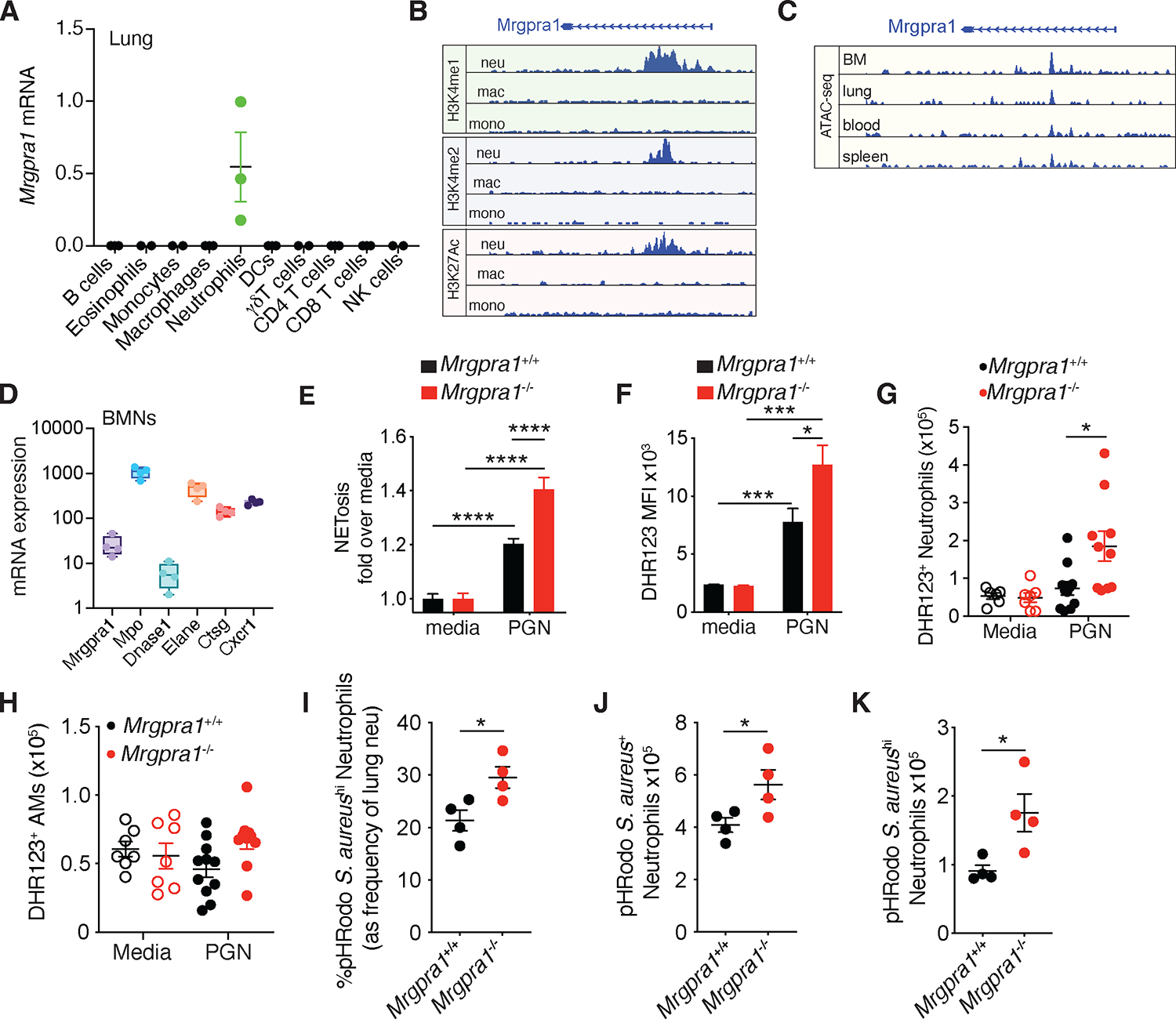
Mrgpra1 is a neutrophil-expressed Mrgpr that regulates their function. (A) *Mrgpra1* mRNA expression relative to *Rps14* mRNA housekeeping gene in flow-sorted B cells (CD11b^−^CD11c^−^CD3^−^gdTCR^−^CD4^−^CD8^−^NK1.1^−^Gr1^−^CD45^+^CD19^+^), eosinophils (CD45^+^ CD11b^hi^ Ly6G^−^ Siglec-F^+^), Ly6C^+^ monocytes (CD45^+^CD11b^hi^Ly6G^−^Siglec-F^−^Ly6C^+^), alveolar macrophages (CD45^+^CD11b^−^CD11c^hi^), neutrophils (CD45^+^CD11b^hi^Ly6G^+^), dendritic cells-DCs (CD45^+^CD11b^+^CD11c^hi^), gamma delta T cells (αβTCR^−^CD4^−^CD8^−^γδTCR^+^), CD4^+^ T cells (αβTCR^+^CD4^+^), CD8^+^ T cells (αβTCR^+^CD8^+^) and NK cells (αβTCR^−^CD4^−^CD8^−^gdTCR^−^NK1.1^+^NKp46^+^). Lungs cells from C57BL/6 mice were pooled (n=4) and sorted for PCR, each dot represents a technical replicate and repeated twice. (B) Histone-seq (GSE63341) of mouse bone marrow-derived neutrophils (BMN) shows peaks for H3K4me1, H3K4Me2, and H3K27Ac at the *Mrgpra1* locus. (C) ATAC-seq of bone marrow (BM), lung, blood, and spleen mouse neutrophils (GSE141285) at the Mrgpra1 locus. (D) Expression of *Mrgpra1*, *Mpo*, *Dnase1*, *Elane*, *Ctsg,* and *Cxcr1* in bone marrow neutrophils. (E,F) Cultured BMNs from *Mrgpra1*^+/+^ and *Mrgpra1*^−/−^ animals (n= 3–4) were treated with media or 50 ug/ml peptidoglycan (PGN) for 4h, after which (E) NETs and (F) ROS were quantified. BMNs from mice (2–3 per group) were plated in replicates and used for experiments. Data is mean+SEM, (G,H) *Mrgpra1*^+/+^ and *Mrgpra1*^−/−^ were given PBS or 200 ug PGN intratracheally (i.t.), and after 3h DHR123^+^ (G) neutrophils and (H) alveolar macrophages (AMs) were enumerated by flow cytometry. Each dot represents an individual animal (n= 4–11), and data are pooled from 2 independent experiments (I-K) Mice were administered pHRodoRed *S. aureus* bioparticles i.t., 2.5h after, the (I) frequency of pHRodoRed *S. aureus*^hi^ neutrophils and numbers of (J) pHRodoRed *S. aureus*^+^ and (K) pHRodoRed *S. aureus*^hi^ neutrophils were analyzed. Data representative of at least 2–3 independent experiments. For all experiments, both male and female mice were used. Data was analyzed by Student’s T test or one-way ANOVA followed by posthoc test. *p<0.05, ***p<0.001, ****p<0.0001. See also Figure S1.

While Mrgprs were initially described as receptors on primary sensory neurons, recent studies have shown Mrgprs to be expressed on immune cells^14^. We aimed to examine the expression of *Mrgpra1*, the founding member of the Mrgprs receptor family, in immune cells. To do this, we extracted RNA from sorted immune cells from naïve mouse lungs. Using real-time PCR to analyze *Mrgpra1* expression, we found it solely present in neutrophils (Fig. 1A). To test whether mast cells expressed Mrgpra1, we used purified peritoneal mast cells, as lung mast cells were technically challenging to sort in large enough numbers to obtain sufficient RNA. We found that mast cells expressed *Mrgprb2* but not *Mrgpra1* (Fig. S1A). To further examine the expression of Mrgpra1 in neutrophils, we looked at various histone modifications associated with active transcription at the *Mrgpra1* locus. In line with our PCR data, ChIP-seq analysis showed enrichment for H3K4me1, H3K4me2, and H3K27Ac in neutrophils but not in macrophages or monocytes (Fig. 1B). Moreover, analysis of ATAC-seq data also revealed an open chromatin configuration for *Mrgpra1* in neutrophils from the bone marrow, lungs, blood, and spleen (Fig. 1C). Consistent with expression in sorted lung neutrophils, we found *Mrgpra1* mRNA expression from RNA-seq of bone marrow neutrophils (BMN) (Fig. 1D). In addition to lung neutrophils, we also find that Mrgpra1 is expressed by liver and spleen neutrophils and not macrophages. While Mrgpra1 is not expressed by lung and liver DCs, it is expressed in spleen DCs. This is in line with others^22^ showing subsets of DCs also express Mrgpra1 (Fig. S1B). In sum, *Mrgpra1* is constitutively transcribed in neutrophils but not in other cells of the lungs’ innate and adaptive immune system.

Next, we investigated the role of Mrgpra1 in the modulation of neutrophil function. To this end, we harvested BMNs (> 90% purity, Fig. S1C) from *Mrgpra1*^+/+^ *and* Mrgpra*1*^−/−^ mice for cellular assays. BMN isolated from *Mrgpra1*^−/−^ animals produced significantly higher level of neutrophil extracellular traps (NET) in response to bacterial peptidoglycan (PGN) compared to *Mrgpra1*^+/+^ controls (Fig. 1E). *Mrgpra1*^−/−^ BMNs also generated more reactive oxygen species (ROS) in response to PGN stimulation as measured by the ROS indicator dihydrorhodamine 123 (DHR123) (Fig. 1F). Consistent with our *in vitro* data, intratracheal (i.t.) administration of PGN resulted in a greater accumulation of DHR123^+^ neutrophils (Fig. 1G), but not DHR123^+^ alveolar macrophages (AMs) (Fig. 1H) in *Mrgpra1*^−/−^ animals as compared to controls. As ROS formation is central to eliminating many microorganisms and its concentration is related to the amount of phagocytosed microbial cargo^23, 24^, we examined whether Mrgpra1 also regulated microbe uptake. To this end, *Mrgpra1*^−/−^ and control mice were exposed i.t. to *Staphylococcus aureus* (*S. aureus)* particles conjugated to the dye pHRodo, a pH-sensitive dye that fluoresces when internalized. We found that the frequency of *S. aureus*^hi^ neutrophils was significantly higher in *Mrgpra1*^−/−^ mice compared to controls (Fig. 1I and Fig. S1D). We also found a greater number of both S. *aureus*^+^ and *S. aureus*^hi^ neutrophils in the lungs of the *Mrgpra1*^−/−^ mice (Fig. 1J, K), indicating that Mrgpra1 restrains the phagocytic potential of neutrophils. As Mrgpra1 governs neutrophil activation, we wanted to determine if it could impact neutrophil numbers and frequency in various tissue. At steady-state, we found equivalent numbers and frequency of neutrophils in blood, BM, spleen, and lungs (Fig. S1E,F) in both genotypes. These findings suggest that Mrgpra1 signaling plays a role in governing the degree of activation of neutrophils during microbial stimulation.

### Mrgpra1 regulates neutrophil activation in response to pulmonary infection.

**Figure 2.**
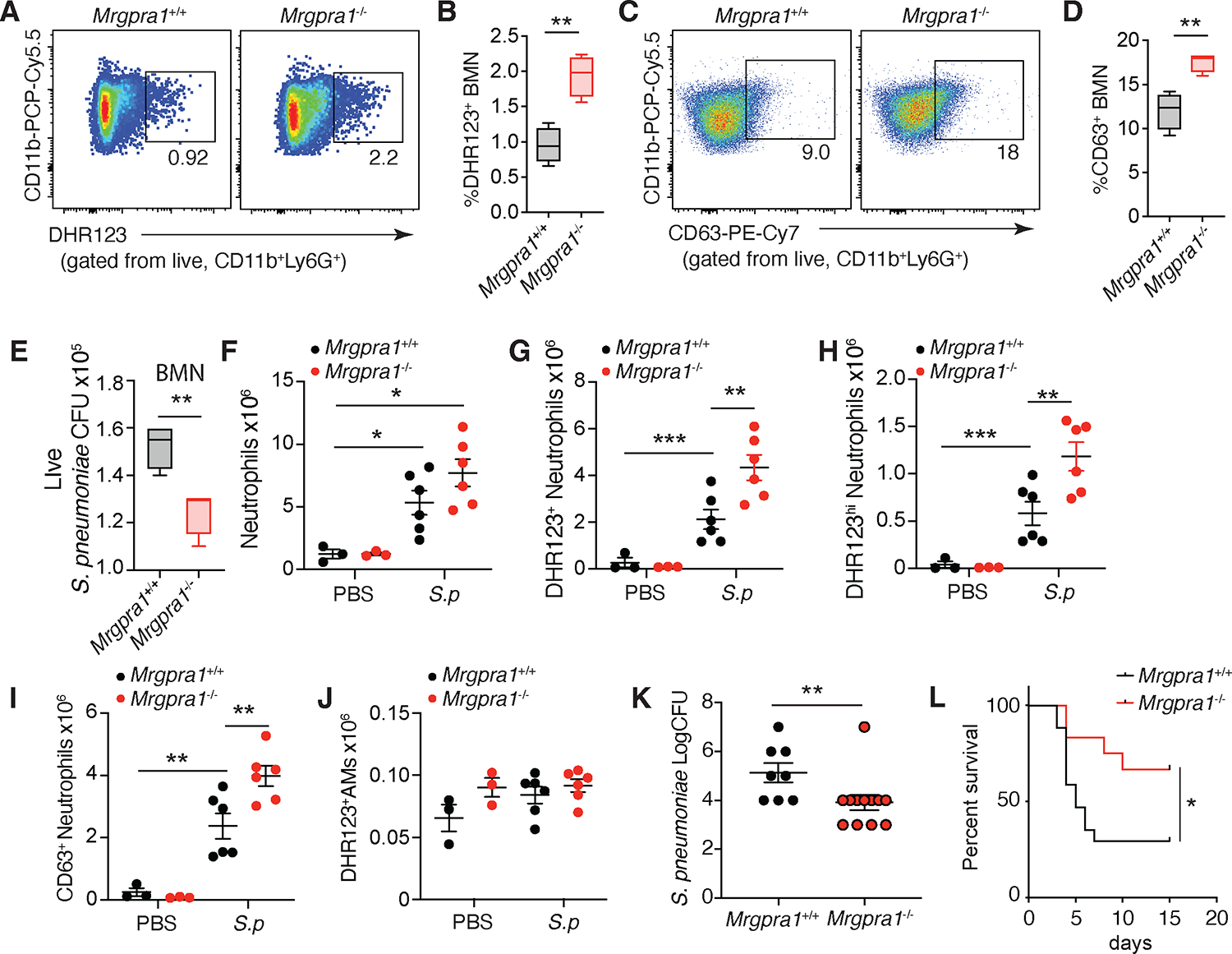
Mrgpra1 signaling dampens neutrophil anti-bacterial responses to *Streptococcus pneumoniae*. (A-E) BMNs from *Mrgpra1*^+/+^ and *Mrgpra1*^−/−^ mice were cultured with *Streptococcus pneumoniae* (MOI 1) for 90 min. Frequency of DHR^+^ (A,B), and CD63^+^ (C,D) neutrophils, and (E) CFU of remaining bacteria. BMNs (pooled) from mice (3–4 per group) were plated in replicates and used for experiments. Data is mean+SEM and representative of at least 2–3 independent experiments. (F-L) *Mrgpra1*^+/+^ and *Mrgpra1*^−/−^ mice given 2.5×10^5^ *Streptococcus pneumoniae* i.t. and 24 h later, the numbers of (F) neutrophils, (G) DHR123^+^ neutrophils, (H) DHR123^hi^ neutrophils, (I) CD63^+^ neutrophils, (J) DHR123^+^ AMs and (K) *S. pneumoniae* CFU in the lungs were quantified. (L) Survival of infected *Mrgpra1*^+/+^ (n=17) and *Mrgpra1*^−/−^ (n=12) mice. Each dot represents an animal (n= 3–12) (F-K). Data is mean+SEM and representative of at least 2–3 independent experiments, or pooled from 2–3 experiments (K,L) For all experiments, both male and female mice were used. Data was analyzed by Student’s T test, one-way ANOVA followed by posthoc test or the Log-rank test for survival *p<0.05, **p<0.01. See also Figure S2.

Our *in vitro* data utilizing microbial surrogates (PGN, *S. aureus* bioparticles) depicted enhanced output by *Mrgpra1*-deficient neutrophils. To further understand this biology in the context of a lung pathogen, we used *Streptococcus pneumoniae (S. pneumoniae)*, a leading cause of bacterial pneumonia. BMNs incubated with opsonized bacteria led to ROS production, but it was significantly more pronounced in *Mrgpra1*^−/−^ than in *Mrgpra1*^+/+^ BMNs (Fig. 2A, B). There was also a greater frequency of *Mrgpra1*^−/−^ neutrophils expressing CD63 (CD63^+^), a marker for degranulation, compared to *Mrgpra1*^+/+^ controls (Fig. 2C, D), suggesting that Mrgpra1 controls ROS production and degranulation of neutrophils. In line with this, significantly fewer bacteria (CFUs) were recovered in co-cultures of *S. pneumoniae* with *Mrgpra1*^−/−^ than with *Mrgpra1*^+/+^ BMNs (Fig. 2E), indicating an elevated functional capacity of *Mrgpra1*^−/−^ neutrophils to kill microbes.

To extend our *in vitro* findings, we ascertained the role of Mrgpra1 in bacterial pneumonia. Mice were administered 2.5×10^5^ CFU of *S. pneumoniae* i.t. 24 h later, lungs were harvested for enumeration of immune cells by flow cytometry (Fig. S2A, flow gating scheme). Infected animals showed an increase in the total number of lung neutrophils, and, while the numbers trended higher in *Mrgpra1*^−/−^ mice as compared to controls, differences did not reach statistical significance (Fig. 2F). In line with our *in vitro* data, the numbers of neutrophils making ROS, as indicated by DHR123^+^ and DHR123^hi^ neutrophils, were higher in *Mrgpra1*^−/−^ mice than in controls (Fig. 2G, H). Further, we found a higher number of CD63^+^ neutrophils in *S. pneumoniae*-infected *Mrgpra1*^−/−^ mice as compared to controls (Fig. 2I). In contrast, we did not observe Mrgpra1-dependent regulation of ROS (DHR123^+^) in alveolar macrophages (AMs) (Fig. 2J). Moreover, the numbers of total and DHR123^+^ Ly6C^hi^ monocytes, and interstitial macrophages (IMs) were not different between *Mrgpra1*^−/−^ and *Mrgpra1*^+/+^ mice exposed to *S. pneumoniae* (Fig. S2 B–D). Nonetheless, in line with elevated ROS^+^ neutrophil numbers, pulmonary *S. pneumoniae* CFUs were significantly reduced in *Mrgpra1*^−/−^ mice compared to controls (Fig. 2K). Finally, we considered the possibility that Mrgpra1 might become expressed on other immune cells in response to infection. To test this, we analyzed *Mrgpra1* expression in various immune cells sorted from *S. pneumoniae*-exposed lungs to test this. Similar to uninfected animals, we found that its expression remained exclusive to neutrophils (Fig. S2F). Notably, the overall enhanced anti-bacterial function of *Mrgpra1*^−/−^ neutrophils resulted in improved survival of *Mrgpra1*^−/−^ mice compared to controls (Fig. 2L).

To examine whether the enhanced functional output by *Mrgpra1*-deficient neutrophils could extend to another pulmonary pathogen, *Mrgpra1*^−/−^ mice were challenged i.t. with *Pseudomonas aeruginosa,* an opportunistic gram-negative bacterium. As with *S. pneumoniae*, we observed a higher number of neutrophils in the lungs of *Mrgpra1*^−/−^ mice and a significantly increased number of ROS (DHR123^+^) producing neutrophils than in controls (Fig. S2 G–H).

### Mrgpra1 signaling balances neutrophil polarization during bacterial infection

**Figure 3.**
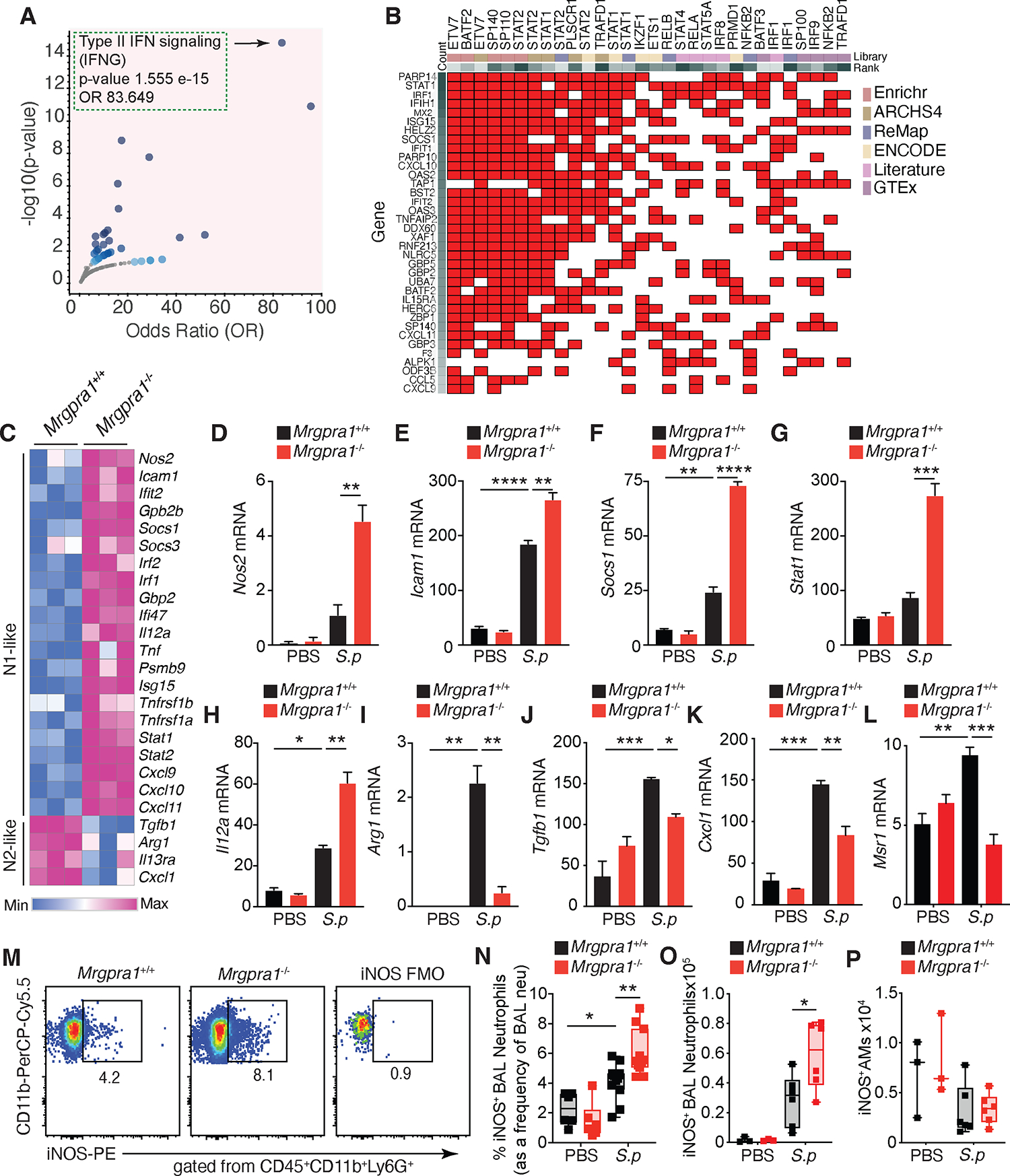
Mrgpra1 signaling balances polarization during bacterial infection (A to L) Male *Mrgpra1*^+/+^ and *Mrgpra1*^−/−^ mice given PBS or 2.5×10^5^ *S. pneumoniae* (*S.p.*) i.t. 24 h after lung neutrophils were flow-sorted for bulk RNA-seq. (A) Plot of enriched pathways in *Mrgpra1*^−/−^ compared to *Mrgpra1*^+/+^ lung neutrophils from infected lungs. (B) Clustergram of TFs that regulate the top expressed genes in *Mrgpra1*^−/−^ lung neutrophils from infected mice using ChIP-X Enrichment Analysis Version 3 (ChEA3). (C) heatmap of genes associated with N1-like and N2-like states in *Mrgpra1*^+/+^ and *Mrgpra1*^−/−^ neutrophils from *S. pneumoniae*-treated mice. (D to L) Normalized expression (transcripts per million) of (D) *Nos2*, (E) *Icam1*, (F) *Socs1*, (G) *Stat1*, (H) *Il12a*, (I) *Arg1*, (J) *Tgfb1* and (K) *Cxcl1* and (L) *Msr1* mRNA. (M-P) *Mrgpra1*^+/+^ and *Mrgpra1*^−/−^ mice given PBS or 2.5×10^5^ *S. pneumoniae* (*S.p.*) i.t. 24h after, BAL was analyzed. (M) Flow plots of iNOS^+^ BAL neutrophils. (N) Frequency of iNOS^+^ BAL neutrophils. (O) Numbers of iNOS^+^ BAL neutrophils. (P) Numbers of iNOS^+^ AMs. Data is mean+SEM, each dot (N-P) represents an animal (n= 3–6) and data is pooled (N) or representative (O,P) from at least 2 independent experiments. For all experiments, both male and female mice were used and data was analyzed by one-way ANOVA followed by posthoc test. *p<0.05, **p<0.01, ***p<0.001, ****p<0.0001. See also Figure S3, Table S1 and Table S2.

To identify the molecular mechanisms downstream of Mrgpra1 that regulate neutrophil function, we performed RNA-seq on flow-sorted lung neutrophils from mice infected with *S. pneumoniae* (sort purity, Fig. S3A). We used the Enrichr Volcano Plot to visualize enriched pathways in *Mrgpra1*^−/−^ lung neutrophils from *S. pneumoniae*-infected mice. This plot shows the significance of each gene set versus its odds ratio, where each point represents a single gene set. This analysis revealed that *Mrgpra1*^−/−^ neutrophils are enriched for genes associated with type II IFNγ signaling, thus suggesting that aberrant IFNγ signaling may drive the enhanced anti-bacterial function of *Mrgpra1*^−/−^ neutrophils (Fig. 3A and Suppl. Tables 1–2).

Further, to understand the molecular pathways that underlie signaling downstream of *Mrgpra1,* we examined the transcription factors (TFs) regulating the activation status of these neutrophils using ChIP-X Enrichment Analysis Version 3 (ChEA3)^25^. This approach obtains the signature of specific TFs in the patterns of differentially expressed genes based on mouse and human data from ENCODE, ReMap, ARCHS4, GTEx, Enrichr, and curated data from the literature^25^. We plotted upregulated genes in *Mrgpra1*^−/−^, as compared to control neutrophils, against the TFs that regulate them. We found a signature (red squares indicate a relationship between gene and TF, white squares indicate no relationship was found) of enriched TFs, such as SP140, BATF2, STAT1, and STAT2 (Fig. 3B), associated with cells polarized to a classically activated phenotype^26–30^. This initial analysis suggested that Mrgpra1 could regulate neutrophil polarization states. We analyzed neutrophils for transcripts associated with IFNγ-mediated polarization, referred to as N(IFNγ) or N1-like neutrophils versus alternatively activated signature genes, denoted as N2-like. We found that neutrophils isolated from infected *Mrgpra1*^−/−^ lungs were enriched for IFNγ-associated transcripts, including *Nos2* (nitric oxide synthase 2), *Icam1*, *Socs1*, *Stat1,* and *Il12a* (Fig. 3C–H). Whereas alternative activation-associated^31, 32^ genes like *Arg1* (Arginase-1), *Tgfb1, Cxcl1* and *Msr1* (CD204) were significantly lower in *Mrgpra1*^−/−^ neutrophils as compared to controls after *S. pneumoniae* challenge (Fig. 3C, I–L). These data establish that Mrgpra1 drives a polarized state compatible with anti-inflammatory alternative activation during bacterial infection.

Next, we explored the role of Mrgpra1 in conditioning differential neutrophil states during infection. To do this, we used the expression of iNOS and Arginase-1 (Arg-1) as indicators of polarized states, where iNOS is associated with N(IFNγ) and Arg-1 is associated with alternative activation. Consistent with our RNA-seq data, we found both a higher frequency and number of iNOS^+^ neutrophils in the BAL of *S. pneumoniae-*infected *Mrgpra1*^−/−^ mice as compared to controls (Fig. 3N–O), but we did not detect differences in iNOS^+^ AMs (Fig. 3P). Moreover, we found fewer Arg1^+^ neutrophils in infected *Mrgpra1*^−/−^ mice’s lungs than controls (Fig. S3B). Because IFNγ-mediated polarization relies on glycolysis and alternative programming upregulates beta-oxidation^33^, we investigated whether Mrgpra1 signaling could regulate the metabolic profile of neutrophils in response to *S. pneumoniae*. We found that *Mrgpra1*^+/+^ neutrophils expressed higher transcripts of beta-oxidation-associated genes (*Acoxl*, *Fabp5*, *Adipor1*, and *Akt2*) as compared to *Mrgpra1*^−/−^ neutrophils (Fig. S3 C–F). Conversely, genes involved in glycolysis, such as *Slc2a1*, *Hk1*, *Hk2*, and *Pfkp,* were upregulated in *Mrgpra1*^−/−^ neutrophils (Fig. S3 C–F).

### Mrgpra1 promotes alternative activation that inhibits IFNγ programming

**Figure 4.**
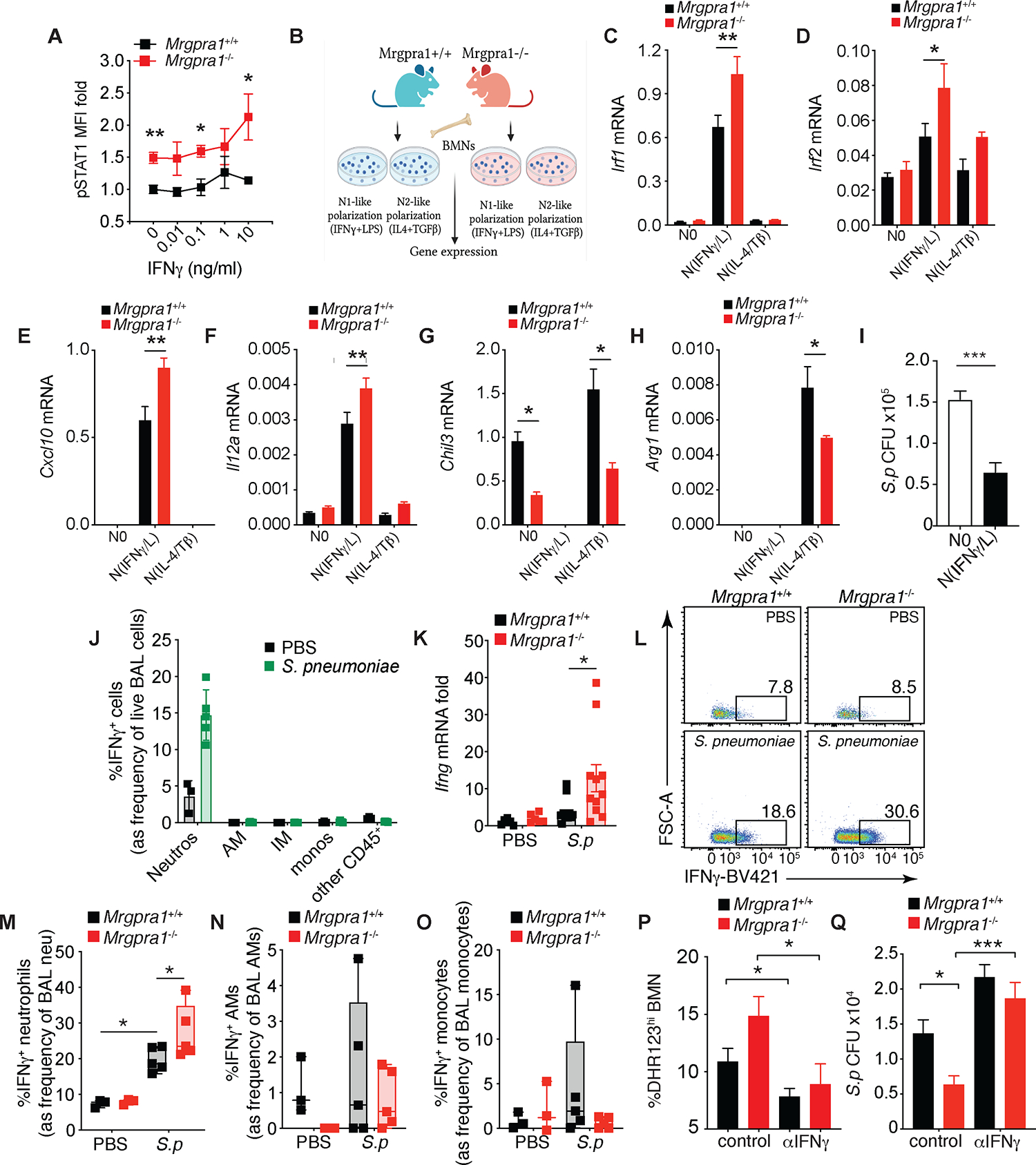
Mrgpra1 promotes alternative activation that inhibits IFNγ programming. (A) phospho-STAT1 MFI in BMNs cultured with increasing IFNγ concentration for 40 mins, data is fold over media. (B-H) (B) BMNs from mice (n=3–4) plated in replicates and cultured in unstimulated N0, N (IFNγ/LPS) or N (IL-4/TGFβ) polarization conditions for 3 h. mRNA of (C) *Irf1*, (D) *Irf2*, (E) *Cxcl10*, (F) *Il12a*, (G) *Chil3,* and (H) *Arg1* determined by PCR. (I) *S. p* CFU recovered in non-polarized (N0) and N1 like-polarized BMNs (MOI 1). (J-O) Mice treated with PBS or 2.5×10^5^ *S. pneumoniae* (*S.p*) i.t. and analyzed 24 h post-infection. (J) BAL numbers of IFNγ^+^ cells and (K) mRNA of total lung *Ifng* mRNA. Frequencies of (L,M) BAL IFNγ^+^ neutrophils, (N) BAL IFNγ^+^ AMs and (O) BAL IFNγ^+^ monocytes. (P,Q) *S. pneumoniae*-exposed BMNs (MOI 1) pre-incubated with control or anti-IFNγ mAbs (20 μg/ml). (P) ROS production and (Q) CFU. Data is mean+SEM, each dot represents an individual mouse (n=3–12), and data is representative of 2–3 independent experiments (C-I and M-Q) or pooled (A,J,O) from 2 independent experiments. For all experiments, both male and female mice were used. Data is analyzed by Student’s T test or one-way ANOVA followed by posthoc test. *p<0.05, **p<0.01, ***p<0.001. See also Figure S4.

Our RNAseq data showed that Mrgpra1 controlled IFNγ-mediated signaling in neutrophils. Because STAT1 is a central mediator of the genes driven by IFNγ, we tested whether Mrgpra1 regulated its activation. We found that at baseline, *Mrgpra1*^−/−^ BMNs have more phospho-STAT1 than controls, suggesting that Mrgpra1 signaling may prevent IFNγ-induced priming (Fig. 4A). Stimulation with rIFNγ also induced greater phospho-STAT1 activation in *Mrgpra1*^−/−^ BMNs as compared to controls (Fig. 4A). In line with this, we found greater expression of *Ifngr1*, but not *Ifngr2* mRNA, in *Mrgpra1*^−/−^ BMNs as compared to wildtype (Fig. S4A,B) and consistently, there was a greater percentage of IFNγR1^+^ neutrophils, but not macrophages or DCs, in the BM of *Mrgpra1*^−/−^ mice as compared to controls (Fig. S4C,D). Based on this we conceived that under defined polarization signals, Mrgpra1 would regulate neutrophil polarization. BMNs were cultured in IFNγ+LPS (to generate N1-like cells), or IL-4+TGFβ1 (to induce N2-like cells) conditions (Fig. 4B), as previously described^1^, and transcript of genes associated with polarized states were analyzed. N(IFNγ+LPS) BMNs were enriched for *Irf1, Irf2*, *Cxcl10,* and *Il12a* mRNAs in cells derived from WT animals (Fig. 4C–F), while N(IL-4+TGFβ1) BMNs expressed *Chil3* and *Arg1* (Fig. 4G–H). As expected, IFNγ+LPS-induced transcripts are not enriched under IL-4+TGFβ1-induced polarization conditions and vice versa (Fig. 4C–H).

In agreement with our *in vivo* data that shows that Mrgpra1 prevents an IFNγ-induced state (see Fig. 3), *Mrgpra1*^−/−^ neutrophils stimulated with IFNγ+LPS expressed more N1-like transcripts than controls (Fig. 4C–F). Conversely, the expression of genes associated with alternative activation was lower in *Mrgpra1*^−/−^ neutrophils stimulated with IL-4+TGFβ1 than in controls (Fig. 4G–H). Moreover, consistent with the notion that IFNγ-conditioned N1-like neutrophils have enhanced anti-microbial properties,^1^ we found that they had greater bacterial killing capacity than non-polarized (N0) neutrophils (Fig. 4I). These data show that Mrgpra1-mediated signaling dampens classical IFNγ-induced programming and promotes an alternatively activated state.

Our data demonstrated that Mrgpra1 inhibited IFNγ signaling, resulting in a reduced anti-bacterial N1-like phenotype. We wanted to know if, besides IFNγ responsiveness, Mrgpra1 could also prevent IFNγ production, as *S. pneumoniae* infection can drive IFNγ production in neutrophils^34^. To this end, mice were infected with 2.5×10^5^ *S. pneumoniae* i.t., and lungs were collected 24 h later to evaluate IFNγ production by flow cytometry. Among all the immune cells examined in the BAL, neutrophils were the predominant source of IFNγ after infection (Fig. 4J). This observation is consistent with others who report that neutrophils are a predominant source of IFNγ upon acute lung infection^35^. When we looked at the role of Mrgpra1, we found significantly higher *Ifng* transcript (Fig. 4K) and IFNγ^+^ neutrophils (Fig. 4L,M) in the lungs of infected *Mrgpra1*-deficient mice as compared to controls. We did not observe changes in IFNγ^+^ macrophages or monocytes in response to infection (Fig. 4N, O). Thus, Mrgpra1 signaling limits IFNγ responsiveness and production, specifically in neutrophils. Finally, we explored the mechanistic contribution of *S. pneumoniae*-induced IFNγ in driving neutrophil activation and bacterial clearance. BMNs from *Mrgpra1*^+/+^ and *Mrgpra1*^−/−^ were pre-treated with IFNγ-neutralizing or isotype control mAb, followed by exposure with *S. pneumoniae*. Robust ROS production was observed in *Mrgpra1*-deficient neutrophils from isotype control-treated animals. However, this was abolished by anti-IFNγ treatment (Fig. 4P). In line with this, neutralizing IFNγ led to a decrease in bacterial killing by *Mrgpra1*-deficient neutrophils (Fig. 4Q). Thus, these data demonstrate that the Mrgpra1 signaling controls the degree of IFNγ-dependent anti-microbial functions of neutrophils.

Our data strongly indicate that neutrophil-intrinsic Mrgpra1 signaling directly inhibits IFNγ mediated polarization. However, because Mrgprs, including Mrgpra1^16^, were traditionally found in sensory neurons, we wanted to ascertain that our phenotype was due to its neutrophil expression and not neuronal participation. Since the lungs are mainly innervated via the vagal ganglia (VG)^36^, we examined whether *Mrgpra1* was expressed in the VG. We found clusters of *Mrgprd*^+^ and *Mrgpra3*^+^ neurons but not of *Mrgpra1*^+^ (Fig. S4E–H). Neurons from the dorsal root ganglia (DRG) make a minor contribution to lung afferents and *Mrgpra1* is expressed in skin DRG neurons^16^. We performed bone marrow chimeras to exclude the possibility that lung innervating Mrgpra1^+^ DRG could participate in our phenotype. Lethally-irradiated *Mrgpra1*^−/−^ hosts were supplemented with either *Mrgpra1*^−/−^ or *Mrgpra1*^+/+^ bone marrow (Fig. S4I). Chimeric animals were exposed to *S. pneumoniae* and 24h post-infection, neutrophils were analyzed. In line with our previous data, numbers of iNOS^+^, IFNγ^+^ and CD63^+^ BAL neutrophils (Fig. S4J–L) were significantly higher in *Mrgpra1*^−/−^ as compared to *Mrgpra1*^+/+^ chimeras. Together, these data reveal a key role for neutrophil-expressed Mrgpra1 in controlling the elaboration of anti-bacterial neutrophils.

### A distinct population of neutrophils express NPFF, the Mrgpra1 ligand, and associates with an alternative phenotype

**Figure 5:**
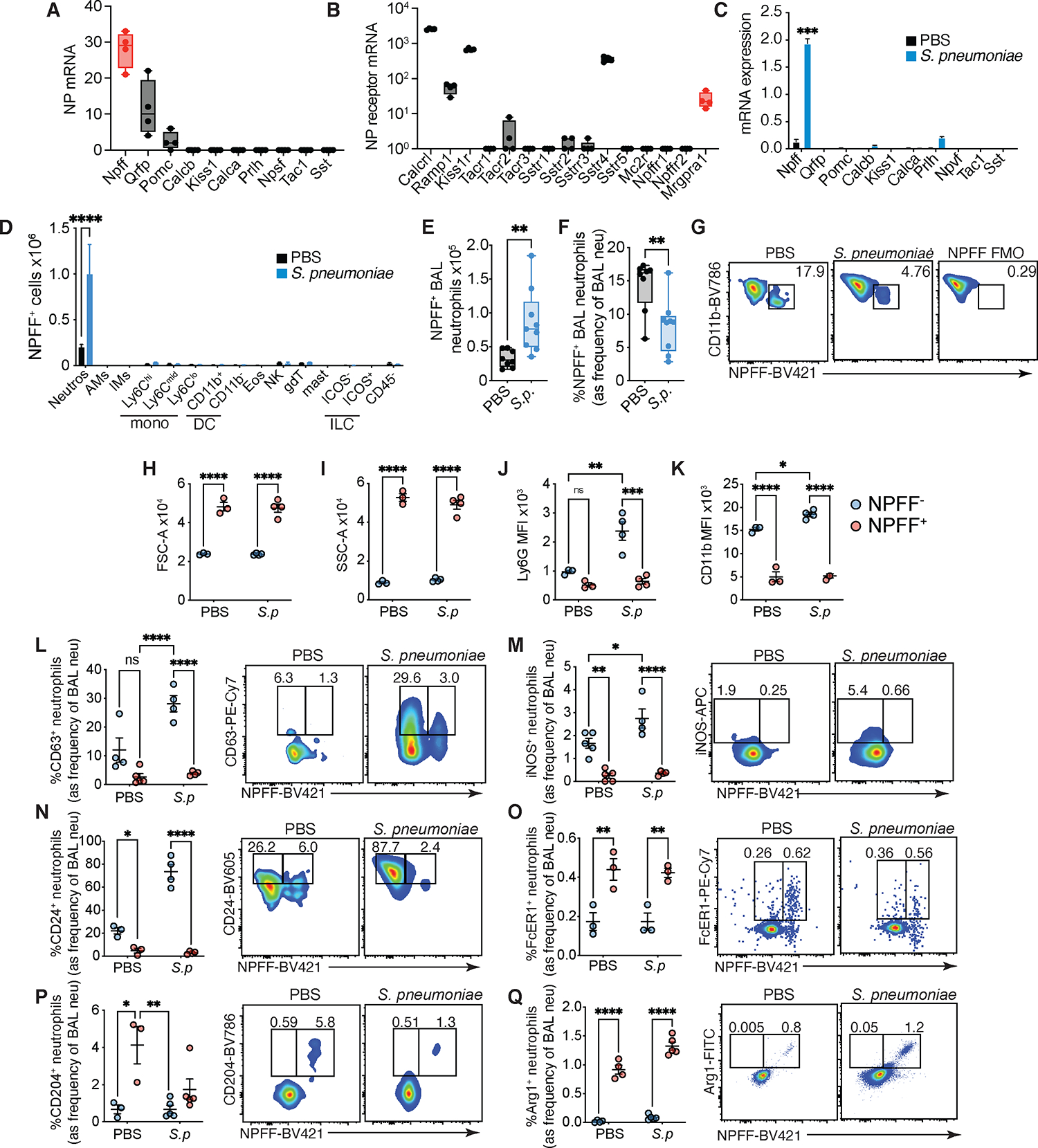
A distinct population of neutrophils express NPFF, the Mrgpra1 ligand, and this populations correlates with an alternative program. (A,B) RNA-seq data (GSE164766) showing expression of (A) neuropeptides (NP) and (B) NP receptors in BMNs. (C) NP mRNAs from BMNs exposed *in vitro* to PBS or *S. pneumoniae* (*S.p*) (MOI 1) for 16 h determined by PCR. (D-G) Mice treated with saline or *S. pneumoniae* and 24 h later (D) number of various NPFF^+^ lung cells, (E) number of NPFF^+^ neutrophils in the BAL and (F) frequency of NPFF^+^ neutrophils. (G) Representative flow plots depicting NPFF staining in BAL neutrophils. (H-Q) Characteristics of NPFF^−^ and NPFF^+^ BAL neutrophils 24h after saline or *S. pneumoniae* (*S.p*) exposure. (H) forward scatter, (I) side scatter, (J) Ly6G MFI and (K) CD11b MFI. Frequency and representative flow plots of (L) CD63^+^, (M) iNOS^+^, (N) CD24^+^, (O) FcεRI^+^, (P) CD204^+^, (Q) Arg1^+^. Data is mean+SEM, each dot (E-Q) represents individual animal (n=3–9) and is representative (H-Q) or pooled (D-F) from at least 2–4 independent experiments that used male and female mice. Data is analyzed by Student’s T test or two-way ANOVA followed by posthoc test. *p<0.05, **p<0.01, ***p<0.001, ***p<0.001. See also Figure S5.

Mrgpra1 is a receptor for several neuropeptides^15^. These include RFamide neuropeptide family members such as the neuropeptide FF (NPFF), somatostatin, and substance P. Among these, NPFF is the most robust mammalian Mrgpra1 ligand^15^, which is generally found in the central nervous system. Because isolated BMN from *Mrgpra1*^−/−^ mice showed phenotypic differences as compared to controls, this suggested that Mrgpra1 could be engaged in a neutrophil-intrinsic manner. Analysis of BMN RNA-seq data showed *Npff* to be a highly-expressed neuropeptide (Fig. 5A).

The canonical receptors for NPFF, *Npffr1* and *Npffr2* were not detected in resting BMNs or after stimulation with *S. pneumoniae* (Fig. 5B and Fig. S5A). We found that in response to *in vitro* exposure to *S. pneumoniae*, *Npff* mRNA was significantly upregulated in neutrophils (Fig. 5C). Next, we explored NPFF^+^ cells *in vivo*, and found that after infection, neutrophils were the predominant source of NPFF^+^ in the lungs (Fig. 5D). In both the lungs (Fig. 5D) and BAL (Fig. 5E), we observed the accumulation of NPFF^+^ neutrophils numbers following infection. While the total number of NPFF^+^ neutrophils increased, owing to greater influx during infection (Fig. 5E), the frequency of neutrophils expressing NPFF decreased after infection, suggesting the release of pre-formed stores of NPFF (Fig. 5F, G).

Next, we wanted to characterize these NPFF+ neutrophils. We found, both at steady state and after infection, that NPFF^+^ neutrophils were larger and more complex than NPFF^−^ neutrophils, as marked by increased forward scatter (FSC, Fig. 5H) and side-scatter (SSC, Fig. 5I), respectively. Ly6G which is dynamically regulated, showed highest expression in activated ROS-producing neutrophils (Fig. S5B,C). In contrast, we found that NPFF^+^ neutrophils were less activated than NPFF^−^ neutrophils as they not only expressed less Ly6G (Fig. 5J) and CD11b (Fig. 5K), but also did not upregulate these markers after infection. Consistent with this, the frequency of degranulating neutrophils (%CD63^+^) was negligible in the NPFF^+^ compared to the NPFF^−^ subset (Fig. 5L). In contrast, CD63 expression was associated with activated ROS^+^ neutrophils (Fig. S5D).

Next, we looked at the expression of markers associated with polarized states. Here we found that NPFF^−^ neutrophils preferentially clustered with IFNγ-associated markers such as iNOS (Fig. 5M) and CD24 (Fig. 5N). However, NPFF^+^ neutrophils preferentially expressed markers associated with an alternative N2-like phenotype like FcεRI (Fig. 5O), CD204 (Fig. 5P), and Arginase-1 (Fig. 5Q). Conversely, CD204, FcεR1 and CD206 were not associated with ROS-producing neutrophils (Fig. S5E–G). Overall, the phenotype of these NPFF-producing neutrophils overlaps with that of alternatively activated cells.

### Mrgpra1-NPFF prevents neutrophil hyperactivation by regulating aberrant IFNγ signaling while promoting an alternatively activated state.

**Figure 6:**
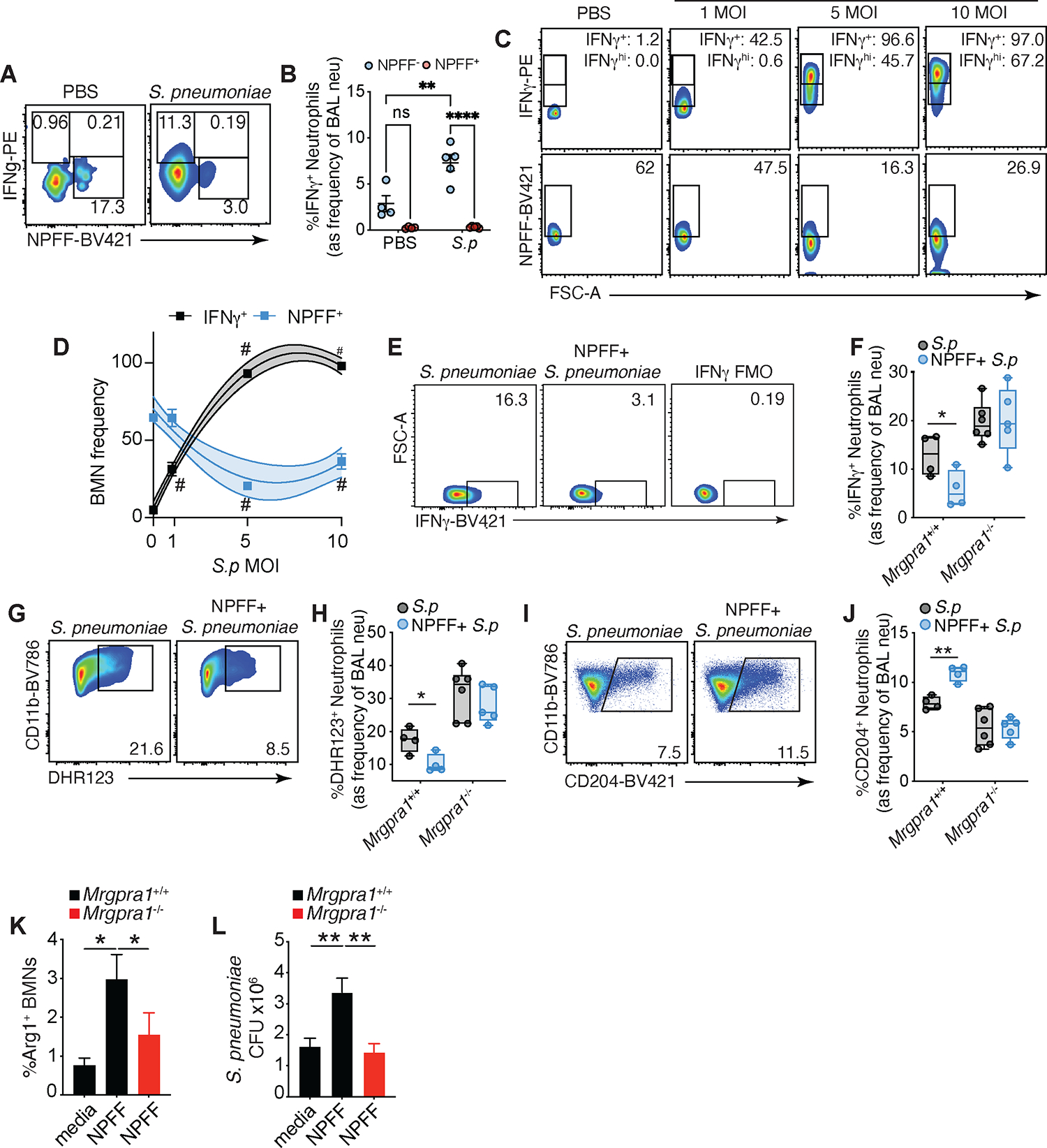
Mrgpra1-NPFF prevents neutrophil hyperactivation by regulating aberrant IFNγ signaling and promotes an anti-inflammatory alternative program. (A,B) (A) Representative flow plots and (B) frequency of IFNγ^+^, in NPFF^−^ and NPFF^+^ BAL neutrophils from saline or *S. pneumoniae (S.p)*-exposed mice. (C,D) (C) Representative flow plots and (D) frequencies of IFNγ, and NPFF staining in BMNs exposed to PBS or *S. pneumoniae* for 4 h (dashed lines represent 95% CI). (E-J) *Mrgpra1*^+/+^ and *Mrgpra1*^−/−^ mice treated with NPFF followed by 2.5×10^5^ *S. pneumoniae* i.t, 24 h post-infection. Frequency of (E, F) IFNγ^+^ neutrophils, (G, H) DHR123^+^ and (I, J) CD204^+^ BAL neutrophils. (K) Percentage of Arg1^+^ BMNs from *Mrgpra1*^+/+^ and *Mrgpra1*^−/−^ mice treated with media or NPFF. (L) CFU from BMNs treated with media or NPFF and incubated with *S. pneumoniae* (MOI 10). Data is mean+SEM and each dot (E-J) represent an animal (n=4–6) and data is pooled (D, K, L) or representative (A-C and E-J) of 2–3 independent experiment using male and female mice. Data and analyzed by Student’s T test or one-way or two-way ANOVA followed by posthoc test. *p<0.05, **p<0.01, #p<0.0001. See also Figure S6.

As our findings indicated that Mrgpra1 signaling regulates IFNγ mediated neutrophil polarization, we next assessed the relationship between NPFF and IFNγ. Analysis of NPFF^+^ and IFNγ^+^ BAL neutrophils showed that they clustered into separate populations at steady state and after infection (Fig. 6A). As we had seen before (see Figs. 4J and 5F), post-infection, neutrophils gained IFNγ and lost NPFF *in vivo* (Fig. 6A). Further, we found that IFNγ^+^ neutrophils were enriched in the NPFF^−^ subset (Fig. 6B). This phenomenon was recapitulated in isolated neutrophils. BMNs incubated with increasing concentrations of *S. pneumoniae* showed decreasing NPFF positivity and increasing IFNγ^+^ cells (Fig. 6C, D). We then tested whether NPFF could directly inhibit the IFNγ phenotype. We found that IFNγ-induced phospho-STAT1 could be inhibited by addition of recombinant NPFF (Fig. S6A). These findings show that NPFF^+^ neutrophils exist as a distinctively regulated population, where a decline in NPFF^+^ neutrophils is concurrent with the elaboration of an IFNγ phenotype.

Next, we wanted to know whether the Mrgpra1-NPFF axis could direct the activation and polarization of neutrophils during infection. To this end, mice were treated with recombinant NPFF, after which they received *S. pneumoniae*. We found that NPFF inhibited infection-induced BAL IFNγ^+^ neutrophils (Fig. 6E, F), and reduced the frequency of ROS^+^ neutrophils (Fig. 6G, H), but not in *Mrgpra1*^−/−^ mice. Conversely, NPFF promoted Mrgpra1-dependent alternative neutrophil polarization, as we measured by an increased frequency of CD204^+^ neutrophils in infected animals (Fig. 6I, J). Lastly, to directly examine the effect of this signaling in neutrophils, we exposed BMNs to *S. pneumoniae* in the presence of NPFF. We found the NPFF-Mrgpra1 axis promoted alternatively activated neutrophils as marked by increased production of arginase-1 (Fig. 6K). Importantly, NPFF conditioning of neutrophils resulted in significant impairment of bacterial clearance in control but not in *Mrgpra1*^−/−^ deficient neutrophil-bacteria co-cultures (Fig. 6L).

In the context of pulmonary bacterial infection, lung-innervating fibers have been shown to play a crucial role in mediating neutrophil function^37^. While NPFF’s neuronal expression is primarily thought to be restricted to the CNS, we looked at whether lung-innervating fibers, which largely emanate from the VG, could express NPFF. While we did not observe clusters of *Npff*^+^ neurons in the VG, as expected we found clusters of *Tac1*^+^ (substance P) neurons (Fig. S6B), which is an Mrgpra1 ligand^22, 38^. This could suggest that SP release from these neurons during infection could regulate neutrophil responses via Mrgpra1. To examine this possibility, we tested the effect of SP on neutrophil-mediated bacteria killing. While we found that NPFF inhibits the ability of neutrophils to kill microbes (Fig.6L), SP pretreatment of neutrophils had no effect on bacterial clearance. As neutrophils express the canonical receptor for SP, TACR1, albeit at low mRNA levels (Fig. S6D), and the EC50 of SP for TACR1 is substantially lower than for Mrgpra1^38, 39^, the net effect of SP on neutrophil will likely be dictated through canonical pathways.

Together, these data demonstrate that a neutrophil intrinsic Mrgpra1-NPFF axis prevents the adoption of an IFNγ-associated phenotype, via control of IFNγ signaling and production. NPFF-mediated regulation of N(IFNγ) cells is concomitant with promoting an alternatively activated program (Fig. 6M). Although the usual host response favors anti-microbial neutrophils, in virulent lung infections, the co-elicitation of N2-like neutrophils would seem detrimental. Our experiments were not designed to experimentally address the potential benefits of alternatively programmed neutrophils during acute infection. However, we speculate that the normal N2-like response may offer advantages in inflammatory resolution and tissue repair in less acutely lethal infections.

## Discussion

Growing evidence shows that Mrgprs regulate functional immunity in immune cells^17–22, 40^. Here we have uncovered a role for neutrophil-expressed Mrgpra1 in regulating their inflammatory and anti-inflammatory polarization states.

The concept that cells undergo a spectrum of activation states is well documented in macrophages. Microbial signals combined with IFNγ elicit M1-like macrophages, also called classically activated or M(IFNγ). They play a microbicidal role through their enhanced production of reactive oxygen and nitrogen species through the enhanced expression of iNOS. M1-like macrophages are thought to be beneficial in clearing bacterial infections^41^. Spectrums of activation states is not limited to macrophages. Recent work demonstrates the existence of similar programs in mouse and human neutrophils^1, 5, 6^. Specifically, N(IFNγ) neutrophils overexpress IFNγ-driven genes, display enhanced ROS generation and are more glycolytic^6^, similar to what is seen in M(IFNγ) macrophages^33, 42^. In line with this, our data demonstrate that neutrophils lacking Mrgpra1 signaling express enhanced IFNγ-associated transcripts, produce themselves more IFNγ, more ROS, and express higher levels of genes involved in glycolysis, ultimately leading to better bacterial clearance. While we observe that Mrgpra1-deficient neutrophils express more IFNγ, they also express more IFNγR1 and downstream signaling molecules. Thus, the aberrant N1-like polarization of Mrgpra1-deficient neutrophils could be in response to IFNγ production alone or in combination with elevated signaling molecules. Mechanistically, blocking IFNγ signaling in neutrophils lacking *Mrgpra1* inhibited the elicitation of ROS and impaired *S. pneumoniae* killing. This suggests that Mrgpra1 regulates IFNγ-dependent neutrophil function during infection. IFNγ signaling is required to protect against *S. pneumoniae*-induced pneumonia^35^, Group A Streptococcal infection^43^ and several other pathogens^44^. Moreover, mice deficient for iNOS, a key IFNγ-induced gene, have impaired lung clearance of *S. pneumoniae*^45^. Our data shows that *Mrgpra1*-deficient neutrophils also express higher transcripts of *Il12a*. IL-12 has been shown to drive *S. pneumoniae* clearance, which occurs through the induction of IFNγ^46^. Consistent with this mouse data, human IL-12 deficiency is associated with chronic *S. pneumoniae* and *S. aureus* infections^47^.

Although beneficial acutely, uncontrolled IFNγ-driven responses can lead to tissue damage^41, 48^ and are associated with greater mortality in patients with sepsis^41^. In LPS-induced acute lung injury (ALI), IFNγ exacerbates pathogenesis.^49^ It is thus conceivable that dysregulated N(IFNγ) could also lead to organ damage Recent studies have shown that during SARS-CoV2 infection, a dysregulated IFNγ-neutrophil axis is associated with mortality and cytokine storm^50, 51^. Thus, mechanisms that counter excessive anti-microbial mechanisms could include eliciting anti-inflammatory, alternatively activated cells, to dampen inflammation by directly inhibiting classical activation or producing anti-inflammatory cytokines like IL-10^33, 41^. In agreement with this, IL-4 confers protection from LPS-induced ALI, partially due to the induction of M2-like, or M(IL-4) macrophages^52^. Similarly, alternatively conditioned neutrophils could be induced to regulate N(IFNγ) responses to resolve inflammation and preserve tissue integrity. Although this may come at the expense of a pathogen clearance efficiency. In line with this, we and others have shown than N2-like cells have less effective anti-bacterial functions. Studies have shown that in humans and mice, IL-4 conditioning of neutrophils renders them ineffective during skin infection with Group A Streptococcus and during *Listeria monocytogenes*-induced sepsis^53–55^. This is thought to partly explain why, in conditions such as atopic dermatitis (AD), where despite greater neutrophils numbers than in healthy individuals, these patients have a higher risk of cutaneous bacterial infections^56^. The type 2 rich environment of AD renders neutrophils compromised in clearing bacteria^56^.

Several neuropeptides (NPs), which are known ligands of Mrgprs^15^, are released during bacterial infections. NPs like vasoactive intestinal polypeptide (VIP), calcitonin gene-related peptide (CGRP), corticotropin-releasing hormone (CRH), and atrial natriuretic peptide (ANP) have been shown to play roles during bacterial infections^37, 57^. Many of these have well-documented anti-inflammatory properties, which may help resolve inflammation^58–60^. However, aberrant production or sensing of these NPs can impair anti-bacterial defenses^37, 57^. The source of NP production during inflammation and infection is typically associated with neurons. Although neurons are thought to be the prototypical source of NPs, they can also be found in non-neuronal cells in the inflamed lungs^61, 62,63–65^. NPs that activate Mrgpra1 include FMRF-amide-related peptides, a family of neuropeptides primarily identified in insects. NPFF, the mammalian homolog of FMRFa^66^, binds Mrgpra1 as well as several other neuropeptides, such as somatostatin and substance P^15, 22, 38^. Notably, our data show that among various neuropeptides, NPFF is expressed by neutrophils and becomes the predominant source of NPFF in response to *S. pneumoniae*. Our data also indicate a mutually exclusive relationship between IFNγ^+^ and NPFF^+^ neutrophils, where these cells form separate clusters. We observe *in vivo* and *in vitro* that neutrophils gain IFNγ production and lose NPFF expression after exposure to bacteria, likely due to neuropeptide release. In comparison to NPFF^−^ neutrophils, when we examined NPFF^+^ neutrophils, we found that they expressed less CD24, CD63 and IFNγ but more Arg1, FcεRI and CD204, suggesting that NPFF^+^ neutrophils are N2-like. Mechanistically, treatment with recombinant NPFF during infection reduced the frequency of IFNγ^+^ and ROS^+^ neutrophils but increased the proportion of CD204^+^ neutrophils and led to a decreased ability by NPFF-conditioned neutrophils to clear bacteria.

Based on our findings, we hypothesize that NPFF-induced neutrophils may adopt an anti-inflammatory phenotype, a well-recognized role for alternatively activated immune cells. This is supported by reports showing that NPFF promotes nerve regeneration and epithelial wound repair in the cornea^67^. Further a recent study identified an IL-4R^+^Arg1^+^CD206^+^Ly6G^lo^ neutrophil subset that promotes nerve regeneration following injury^68^. This neutrophil subset increases in numbers chronically over days following injury. NPFF^+^ N2-like neutrophils are similar to these as they are also Arg1^+^CD206^+^Ly6G^lo^. This highlights the likelihood that NPFF- or IL-4-conditioned neutrophils may accumulate following acute inflammation to promote resolution of inflammation and tissue repair. In addition, our findings are consistent with work in adipose tissue macrophages, where NPFF can inhibit LPS-induced nitric oxide^60^ and induce M2-like polarization in an NPFFR2-dependent fashion^69^. Recent work shows that Mrgpra1 mediates the recruitment of a dendritic cell subset, CD301b^+^ dermal dendritic cell, in the skin in response to papain via a substance P-dependent mechanism^22^, suggesting this Mrgpr may enhance type 2 responses at barrier sites. While we did not find Mrgpra1 in lung and liver DCs, it is expressed in splenic DCs. This highlights that Mrgpra1 expression may be selective to a few cell types in a tissue-specific manner and plays a central role in barrier immunity.

Taken together, our data support a role for an intrinsic Mrgpra1-NPFF axis in regulating a balance between classically activated N1-like or alternatively activated N2-like states. However, we cannot discount the possibility that NPFF or other Mrgpra1 ligands, like substance P and somatostatin, secreted by other cells in the pulmonary tissue, may be induced during infection to modulate neutrophil function. Further, we find that without adding exogenous NPFF, Mrgpra1 signaling promotes IL-4-induced polarization *in vitro*. While this may suggest that IL-4 could induce NPFF in BMNs, another possibility would be the inability of *Mrgpra1*^−/−^ neutrophils to receive NPFF conditioning *in vivo*, resulting in unopposed and lasting IFNγ programming. This is supported by our data where baseline *Mrgpra1*^−/−^ BMNs express lower transcripts of the N2-like marker chitinase (Chil3) and higher phospho-STAT1 than controls. Importantly, beyond the established drivers of alternative activation, such as IL-4, IL-13, IL-10 and TGFβ, we have found that NPFF can polarize neutrophils to an alternative N2-like state. Therefore, we suggest that NPFF acts as a non-canonical driver of an alternative program. Because at steady state, we detect NPFF^+^ neutrophils in the airways and in the bone marrow, NPFF may provide homeostatic signals.

Our findings likely extend beyond bacterial infections, as dysregulated states of neutrophil activation is now thought to play a pathogenic role in various inflammatory diseases such as cancer, stroke, and myocardial infarctions^2, 3, 5, 70^. In sum, we have identified a pathway that balances neutrophil responses, which may serve as a target for preventing and treating refractory lung infections.

### Limitation of the Study

Our studies were designed to study the acute response by neutrophils post-infection. However, it remains to be determined the role of Mrgprs and neuropeptides in regulating the extent of tissue damage and to evaluate their role in resolution of inflammation in chronic settings. Moreover, while we focused on *S. pneumoniae*, other common pneumonia-causing bacterial pathogens like *Klebsiella pneumoniae*, *S. aureus*, and mycoplasma, remain to be studied. Further, arginase-1 secretion from neutrophils causes T cell immunosuppression^71^, therefore, it remains a possibility that this NPFF pathway participates in the development of subsequent adaptive immune responses to pathogens. Moreover, while Mrgpra1 remains exclusive to lung neutrophils during infection, we anticipate that during sepsis, Mrgpra1 on other immune cells like dendritic cells may get engaged in lymphoid organs like the spleen and contribute to host responses. Future studies using cell-specific deletion of Mrgpra1 would be valuable to evaluate its contribution on different cells. Lastly, as alternatively activated neutrophils exist in humans, we posit that our findings will extend to human neutrophils. Future studies will be done to evaluate this pathway in humans.

### Resource availability

#### Lead contact

Further information and requests for resources and reagents should be directed to and will be fulfilled by the lead contact, Xinzhong Dong (xdong2@jhmi.edu).

#### Materials availability

This study did not generate new unique reagents.

#### Data and code availability

- RNA-seq data from sorted lung neutrophils are deposited in the Gene Expression Omnibus (GEO) database and are publicly available from the date of publication Accession numbers are listed in the Key Resources Table. The following public datasets were used: Histone ChIP-seq dataset from mouse bone marrow neutrophils, bone marrow monocytes, and peritoneal macrophages; ATAC-seq datasets of mouse neutrophils; RNAseq from bone marrow neutrophils (BMN; and Single cell RNAseq of mouse vagal ganglia. Accession numbers are listed in the Key Resources Table.
- This paper does not report original code.
- Any additional information required to reanalyze the data reported in this paper is available from the lead contact upon request.

### Experimental model and study participant details

#### Mice

*Mrgpra1*-deficient mice were generated as previously described^76^. Briefly, *Mrgpra1-*deficient animals were generated on 129 genetic background. Mrgpra1 locus (exon 2) on mouse Chromosome 7 was targeted via homologous recombination. Age (5–12 weeks) and sex-matched (males and females) *Mrgpra1*^−/−^ and *Mrgpra1*^+/+^ littermate mice were used for all experiments. For studies requiring intranasal challenges, mice were briefly anesthetized using isoflurane. For intratracheal administrations (i.t.), mice were anesthetized with intraperitoneal administration of ketamine-xylazine solution. All mice were maintained in a specific-pathogen free facility and used according to the Institutional Animal Care and Use Committee.

### Method details

#### Flow cytometry

Mouse lung and, spleen cells were obtained following digestion with 0.05 mg/ml Liberase TL (Roche) and 0.5 mg/ml DNaseI (Sigma) for 45 min at 37°C in 5% CO_2_. Liver single cell suspension were obtained as described^84^. Digested tissue was filtered through a 70-um nylon mesh (BD Biosciences). Lung, spleen, liver, bone marrow and blood-derived cells were suspended in red blood cell lysis buffer (ACK lysis buffer). Recovered cells were counted (trypan blue exclusion) and plated at 1–2 ×10^6^ cells/ml. Cells were washed with PBS and labeled with Zombie Aqua live/dead dye (Biolegend) for 10 min at RT and blocked with 10 ug/ml anti-CD16/32 (BioLegend or BioXCell) for an additional 20 min at RT. Cells were stained with fluorochrome-labeled antibodies. For intracellular staining, cells were first fixed with 4% paraformaldehyde for 10 mins at RT, then permeabilized with 0.1% saponin for 20 min at RT. For NPFF intracellular staining, fixed and permeabilized cells were stained with a primary anti-NPFF antibody (30 min), followed by two washes with permeabilization buffer and then stained for BV421-labelled secondary antibody (30 min). NPFF FMO cells were stained with BV421-labeled secondary antibody without anti-NPFF primary. Data were acquired on an LSRII flow cytometer (BD Biosciences) and gated to exclude debris and select single cells (SSC-W/SSC-A). Data were analyzed using FlowJo (BD Biosciences).

#### PGN and *S. aureus* bioparticles administrations

Mice were given saline or 200 μg *Staphylococcus aureus*-derived peptidoglycan (PGN-SA) (Invivogen) intratracheally (i.t.), and 3 h later, lungs were harvested for flow cytometry. In other experiments, 80 μg pHrodoRed-labeled *S. aureus* bioparticles (Thermo) were given i.t. and 2.5 h later, lungs were harvested and cells isolated for flow cytometry.

#### Bacterial cultures

*Streptococcus pneumoniae* 6303 (ATCC) was grown and maintained on sheep blood agar (BD Biosciences). Single colonies were harvested into PBS, followed by serial dilution plating to determine bacterial stock concentration. *Pseudomonas aeruginosa (PAO1)* was grown in tryptic soy broth (Sigma-Aldrich) overnight in a shaking incubator (37°C; 150 rpm). An aliquot of stock was removed, and pellets were washed thrice with PBS. Serial dilutions were then plated onto tryptic soy agar plates (Teknova) to determine stock concentrations.

#### Opsonization of *S. pneumoniae*

*S. pneumoniae* were mixed in 1:1 volume (50% opsonization) with normal mouse serum (Invitrogen) and incubated at 37°C for 30 min, shaking at 230 RPMs. Bacteria were washed twice in PBS and suspended in RPMI containing 10% FBS and 2 mM L-glutamine, without penicillin/streptomycin.

#### *In vivo* bacteria administration

For experiments with *S. pneumoniae,* 2.5×10^5^ CFU were administered (40 μl in PBS) i.t., for *Pseudomonas aeruginosa,* mice were exposed to 10^7^ CFU i.t. (40 μl in PBS). In some experiments, mice were given PBS or 4 nmoles NPFF (Tocris) intranasally, 24 h later, mice were given 2.5×10^5^ *S. pneumoniae* CFU, with or without NPFF, intratracheally. For IFNγ blockade, mice received isotype control or 0.2 mg anti-IFNγ (XMG1.2, BioXCell) in 100 ul of PBS intraperitoneally a day before infection.

#### Bone marrow chimeras

Bone marrow chimeras were performed as described previously^85^. Briefly, 10- to 12-week-old Mrgpra*1*^−/−^ mice were irradiated with two doses of 4.5 Gy given 3 hours apart. After, mice were injected intravenously (2×10^6^ cells/200 ul) with bone marrow cells from *Mrgpra1*^+/+^ or *Mrgpra1*^−/−^ mice. Mice were kept on neomycin (1.1 mg/ml) water for 2 weeks following irradiation. At the end of 12–15 weeks, mice were challenged with *S. pneumoniae* (2.5 X 10^5^ CFU) intratracheally, and 24h later, BAL was harvested for flow cytometric analysis.

#### Peritoneal mast cell (PMC) culture

PMCs were cultured as described^86^. Briefly, the peritoneal cavity of 2–3 mice was flushed using ice-cold sterile PBS (10 ml each) and the peritoneal cavity was gently massaged. The peritoneal fluid was then collected into a 50 ml conical and cells were spun (300×g, 10 min) followed by a PBS wash. Cells were resuspended in 5 ml complete media (RPMI, 10% FBS/pen/strep) supplemented with recombinant mouse (rm) IL-3 (10 ng/ml) and rmSCF (30 ng/ml). The cell suspension was then transferred to a 25 cm^2^ tissue culture flask and incubated at 37°C, 5% CO_2_. At day 3, non-adherent cells were discarded with the media and fresh media (5 ml) containing rmIL-3 (10 ng/ml) and rmSCF (30 ng/ml) was added. On day 6, 5 ml of fresh media containing rmIL-3 (10 ng/ml) and rmSCF (30 ng/ml) was added to the culture. At the end of the culture (day 10), PMCs which are the floating, non-adherent cells were harvested and sorted to >95% purity (CD45^+^FcεR1^+^c-kit^+^) and cells (1s10^6^ cells) were used for RT-PCR.

#### Bone marrow-derived neutrophil (BMN) isolation

Marrow from tibias and femurs was harvested, and bone-marrow-derived neutrophils (BMN) were isolated using Histopaque (Sigma) employing a protocol described previously^87^. Briefly, bone marrow was collected from mice in PBS containing 2 mM EDTA, followed by red-blood cell lysis with ACK buffer. The bone marrow cells were then suspended in ice-cold PBS and gently layered onto Histopaque to avoid disturbing the gradient. After centrifugation (872×g, 30 min), BMNs were collected at the interface layer and washed twice in RPMI containing 10% FBS, 2 mM L-glutamine, and penicillin/streptomycin. BMNs were seeded at 1.5–2.5×10^5^ cells in round-bottom 96-well plates in RPMI containing 10% FBS, 2 mM L-glutamine, and penicillin/streptomycin. For experiments, BMNs from multiple (n= 3–6) mice were pooled for each genotype and experiments were performed with replicates. We routinely harvest high purity (>90%) BMNs using this isolation method. Further, we obtain similar numbers of neutrophils from both *Mrgpra1*^−/−^ and *Mrgpra1*^+/+^ mice.

#### N1-like/N2-like polarization cultures

BMNs were seeded at 2.5×10^5^ cells in round-bottom 96-well plates in RPMI containing 10% FBS, 2 mM L-glutamine and stimulated with 20 ng/ml IFNγ (RnD Systems) and 1 μg/ml LPS (Sigma) for N1-like or 20 ng/ml IL-4 (RnD Systems) and 10 ng/ml TGFβ1 (Peprotech) for N2-like polarization for 3 h. Following this, cells were harvested and used for RNA isolation. Some BMNs were incubated overnight with 10 uM NPFF (Tocris). For intracellular staining, cells were first fixed with 4% paraformaldehyde for 10 mins at RT, then permeabilized with 0.1% saponin for 20 min at RT, followed by intracellular staining.

#### NET assay

1.5×10^5^ BMNs were resuspended in RPMI containing 10% BSA and plated in black 96-well dishes with clear bottoms and treated with media or 50 μg/ml PGN for approx. 3.5 h. Following this, 1 ug/ml Hoechst (Invitrogen) and 5 μM SYTOX orange (Thermo) was added, and cells were incubated for another 10 min at 37°C with 5% CO_2_. NETosis was assessed as a ratio of fluorescence from SYTOX orange (cell-impermeable nucleic acid stain) to Hoescht (cell-permeable nucleic acid stain).

#### ROS assay

For *in vitro* ROS measurement, 1.5×10^5^ BMNs were resuspended in RPMI containing 10% FBS and 2 mM L-glutamine and plated in round-bottom 96-well dishes. Cells were stimulated with media or 10 μg/ml PGN for 2 h. During the last 20 min of incubation, 0.5 μg/ml DHR123 (Thermo) was added. Cells were washed and analyzed by flow cytometry. For *in vivo* ROS analysis, 2×10^6^ lung cells in 96-well dishes were incubated with 0.5 μg/ml DHR123 for 20 min, after which cells were washed and prepared for flow cytometry.

#### BMN-bacteria cultures

BMNs were seeded at 1.5×10^5^ cells in round-bottom 96-well plates in RPMI containing 10% FBS, 2 mM L-glutamine (without penicillin/streptomycin) in combination with 0.15×10^5^ (ROS measurement). For bacteria killing assay a MOI of 1–10 utilizing opsonized *S. pneumoniae* CFU in a total volume of 200 μl was performed. Plates were incubated at 37°C for 120 min with shaking (180 RPMs). During the last 20 min of incubation, 0.5 μg/ml dihydrorhodamine 123 (DHR123, Thermo) was added. Cells were washed and analyzed by flow cytometry. For CFU enumeration, BMNs and bacteria were incubated as above, and 20 μL (10% of the culture volume) was taken and incubated in 980 μL pH 11 water to lyse neutrophils for 20 min at 37°C. 10 μL of this mixture was used for serial dilution on sheep blood agar plates. Plates were incubated overnight for CFU enumeration. In specific experiments, BMNs were treated with NPFF (25 uM), substance P (25 uM) or in N1-like conditions for 3 h or with 20 μg/ml control or anti-IFNγ mAbs for 40 min before addition of bacteria.

#### Real-time PCR

Total RNA was extracted using TRIzol (Thermo) and cDNA generated (Applied Biosystems). PCR was performed using primer probes (Thermo). For experiments with BMN, 0.5 μg of RNA was used, whereas, for lung tissue, 2 μg of RNA was utilized to prepare cDNA. Data are expressed as relative expression to the housekeeping gene ribosomal protein S14 (*Rps14*).

#### Phosphoflow

BMNs were treated with recombinant mouse IFNγ for 40 mins in RPMI containing 2% FBS with l-glutamine and penicillin/streptomycin. Cells were fixed by directly adding 32% PFA (Electron Microscopy Sciences) for a final concentration of 1.6% for 10 min. PFA was removed, and BMNs were permeabilized with ice-cold 90% methanol for 60 min at 4°C. Cells were stained with phycoerythrin-conjugated phospho-STAT1 antibodies in PBS+0.2% BSA and washed with PBS+0.2% BSA. In some experiments, BMNs were pretreated with NPFF (25 uM) for 4h before the addition of mouse IFNγ for 45 min.

#### Flow sorting for PCR

Lung cells from naïve mice or *S. pneumoniae* infected mice (24 h p.i) were pooled (n=3–4) and stained (see legends of Figure 1 and Fig. S2), and various live (propidium iodide^−^) lung immune cells were flow-sorted using a MoFlo XDP (JHU Cell Sorting Core Facility). Liver and spleen cells from naïve mice (n=3) were pooled for sorting and various populations were sorted as described (see Figure S1 legend). Cells were split into replicates and resuspended in TRIzol for RNA extraction.

#### RNA sequencing of lung neutrophils and analysis

*Mrgpra1*^+/+^ and *Mrgpra1*^−/−^ mice (n=4–5) were treated (i.t.) with saline or 2.5×10^5^ *S. pneumoniae* CFU, 24 h later, lungs were harvested and processed for single-cell suspensions. Neutrophils (propidium iodide^−^CD45^+^CD11b^+^Ly6G^+^) were flow-sorted (see above) to more than 95% purity (Fig. S3a). To mitigate the low quantity of RNA in pulmonary neutrophils (about 20 times less than other cells), neutrophils from 4–5 animals in each group were pooled and then split into replicates prior to RNA extraction. RNA was extracted using RNeasy columns (Qiagen), and genomic DNA was removed by on-column digestion. Library construction and sequencing were done commercially (Cofactor Genomics) using the picoRNA platform. Reads were mapped to GRCm38, and differential expression was performed using DESeq2. Data are expressed as normalized transcripts per million.

#### scRNAseq data analysis

Cells were processed via the Seurat 4.0 workflow. All cells that expressed fewer than 200 genes were removed. Cells with high (>30%) mitochondrial reads were removed. Doublets were removed via the ‘scDblFinder’ method. Datasets were integrated using Harmony and Log-normalized gene expression data was used for visualizations.

#### Quantification and statistical analysis

Statistical significances were calculated using Student’s T test or one-way ANOVA followed by posthoc test, as indicated in figure legends. P-value are denoted as: *p<0.05, ***p<0.001, ****p<0.0001. Error bars on all figures represents standard error of mean (SEM).

## Supplementary Material

1

2 Table S1: Pathway analysis of lung neutrophils depicting odd ratios post-infection. Related to Figure 3.

3 Table S2: List of differentially expressed (DE) genes in Mrgpra1 deficient lung neutrophils post-infection. Related to Figure 3.

## Figures and Tables

**Key resources table:** 

REAGENT or RESOURCE	SOURCE	IDENTIFIER
Antibodies		
anti-mouse CD45-APC-Cy7 (1/800)	BioLegend	Cat#103115; RRID: AB_312980
anti-mouse CD45-Alexa 700 (1/800)	BioLegend	Cat#103127; RRID: AB_493714
anti-mouse Ly6C-PE-Dazzle594 (1/400)	BioLegend	Cat#128043; RRID: AB_256676
anti-mouse Ly6G-APC-Cy7 (1/800)	BioLegend	Cat#127623; RRID: AB_10645331
anti-mouse Ly6G-BV421 (1/800)	BioLegend	Cat#127627; RRID: AB_10897944
anti-mouse CD63-PE-Cy7 (1/300)	BioLegend	Cat#143909; RRID: AB_2565499
anti-mouse CD11c-BV605 (1/400)	BioLegend	Cat#117333; RRID: AB_11204262
anti-mouse CD11b-PE-Cy7 (1/2500)	BioLegend	Cat#101215; RRID: AB_312798
anti-mouse CD11b-PerCP-Cy5.5 (1/2500)	BioLegend	Cat#101227; RRID: AB_893233
anti-mouse CD11b-BV785 (1/2500)	BioLegend	Cat#101243; RRID: AB_2561373
anti-mouse Siglec-F-PerCP-Cy5.5 (1/400)	BD Biosciences	Cat#565526; RRID: AB_2739281
anti-mouse CD64-BV421 (1/150)	BioLegend	Cat#139309; RRID: AB_2562694
anti-mouse MerTK-APC (1/150)	BioLegend	Cat#151507; RRID: AB_2650738
anti-mouse MerTK-PE (1/150)	BioLegend	Cat#151505; RRID: AB_2617036
anti-mouse MerTK-PE-Cy7 (1/150)	BioLegend	Cat#151521; RRID: AB_2876508
anti-mouse IFNγ-BV421 (1/100)	BioLegend	Cat#505829; RRID: AB_10897937
anti-mouse IFNγ-PE (1/100)	BioLegend	Cat#505807; RRID: AB_315401
anti-mouse iNOS-PE (1/100)	Thermo	Cat#12-5920-82; RRID: AB_2572642
anti-mouse Arginase-1-PE (1/100)	Thermo	Cat#12-3697-82; RRID: AB_2734839
anti-mouse pSTAT1-PE (1/100)	Thermo	Cat#MA5-37073; RRID: AB_2897008
anti-mouse CD204-BV421 (1/100)	BD Biosciences	Cat#748083; RRID: AB_2872544
anti-mouse CD204-BV786 (1/100)	BD Biosciences	Cat#748089; RRID: AB_2872550
anti-mouse CD206-BV605 (1/100)	BioLegend	Cat#141721; RRID: AB_2562340
anti-mouse CD24-BV605 (1/300)	BioLegend	Cat#101827; RRID: AB_2563464
anti-mouse ckit-BV421 (1/200)	BioLegend	Cat#135123; RRID: AB_2562236
anti-mouse FcER1-PE-Cy7 (1/200)	BioLegend	Cat#134317; RRID: AB_10643996
anti-mouse FcER1-APC (1/200)	BioLegend	Cat#134315; RRID: AB_10640726
Donkey anti-rabbit IgG-BV421 (1/400)	BioLegend	Cat#406410; RRID: AB_10897810
rabbit anti-NPFF polyclonal (1/200)	Thermo	Cat#PA5-25175; RRID:AB_2542675
anti-mouse IFNγR1-BV605 (1/100)	BD Biosciences	Cat#745111; RRID:AB_2742716
anti-mouse IFNγR2-Ax647 (1/100)	R&D Systems	Cat#FAB773R
TruStain FcX^™^ (anti-mouse CD16/32)	Biolegend	Cat#101319 RRID:AB_1574975
*InVivo*MAb anti-mouse CD16/CD32	BioXcell	Cat# #BE0307 RRID:AB_2736987
anti-mouse IFNγ	BioXcell	Cat#BE0055 RRID:AB_1107692
Bacterial and virus strains		
*Streptococcus pneumoniae* 6303	ATCC	Strain ID: CIP 104225
*Pseudomonas aeruginosa (PAO1)*	Dr. Ying Zhang (JHU)	N/A
PGN from Staphylococcus aureus	InvivoGen	Cat# tlrl-pgns2
pHrodo^™^ Red S. aureus BioParticles^™^	ThermoFisher	Cat# A10010
Chemicals, peptides, and recombinant proteins		
Neuropeptide FF	Tocris	Cat#3137
Substance P	Tocris	Cat# 1156
anti-mouse IFNγ	BioXcell	Cat#BE0055
Recombinant Mouse IL-3	R&D systems	Cat# 403-ML-010
Recombinant Mouse SCF	R&D systems	Cat# 455-MC-010
Recombinant Murine IFNγ	Peprotech	Cat# 315-05
Recombinant Murine IL-4	Peprotech	Cat# 214-14
Recombinant Mouse TGF-beta 1	R&D systems	Cat#: 7666-MB
Histopaque^®^-1119	Millipore Sigma	Cat# 11191
Histopaque^®^-1077	Millipore Sigma	Cat# 10771
SYTOX^™^ Orange Nucleic Acid Stain	ThermoFisher	Cat# S11368
Hoechst 33342	ThermoFisher	Cat# H1399
Normal Mouse Serum	ThermoFisher	Cat# 10410
TRIzol^™^ Reagent	ThermoFisher	Cat# 15596026
32% Paraformaldehyde	FisherScientific	Cat# 50-980-494
Tryptic Soy Agar Plates	Teknova	Cat# T0144
Blood agar plate	BD	Cat# 22126
Penicillin-Streptomycin-Glutamine (100X)	ThermoFisher	Cat# 10378016
Fixation & Permeabilization Buffer set	ThermoFisher	Cat# 88-8824-00
Liberase ^™^ TL	Millipore Sigma	Cat#05401020001
Deoxyribonuclease I	Millipore Sigma	Cat#DN25
Zombie Aqua viability dye	BioLegend	Cat#423101
Dihydrorhodamine 123	ThermoFischer	Cat# D632
Deposited data		
RNAseq of sorted mouse lung neutrophils	this manuscript	Accession# GSE200214
Histone-ChIP (neutrophils, monocytes, macrophages	Lavin et al.^72^	Accession# GSE63341
ATAC-seq of mouse neutrophils	Ballesteros et al.^73^	Accession# GSE141285
RNAseq of mouse bone marrow neutrophils (BMN)	Timaxian et al.^74^	Accession# GSE164766
scRNAseq of mouse vagal ganglia	Zhao et al.^75^	Accession# GSE192987
Experimental models: Cell lines: not applicable		
Experimental models: Organisms/strains		
Mrgpra1^−/−^ mice	Liu et al.^76^	N/A
Oligonucleotides		
mNpff.S: 5’-GGTCCCTCTTTCGTGTTCTG-3’ AS: 5’-GCG GAT TTA GCT GTT CCT TG-3’	Integrated DNA Technologies	N/A
mNpvf.S: 5’-CATGATGCCTCATTTTCACAGC-3’ AS: 5’-CCTCTCCTCGTTCGCTTTCC-3’	Integrated DNA Technologies	N/A
mQrfp: S:5’-TCACCTGCCCTTCTTAGAGC-3’ AS: 5’-CGGTTCAAAATCCACAGCCA-3’	Integrated DNA Technologies	N/A
mSst.S: 5’-CTCCGTCAGTTTCTGCAGAA-3’ AS: 5’-TTCTCTGTCTGGTTGGGCTC-3’	Integrated DNA Technologies	N/A
mTac1.S: 5’-TTTCTCGTTTCCACTCAACTGTT-3’ AS: 5’-GTCTTCGGGCGATTCTCTGC-3’	Integrated DNA Technologies	N/A
mPrlh.S: 5’-CCCTGACATCAATCCTGCCT-3’ AS: 5’-GCTGTGAGAGAACTTGGCAC-3’	Integrated DNA Technologies	N/A
mCalca.S: 5’-CAGTGCCTTTGAGGTCAATCT-3’ AS: 5’-CCAGCAGGCGAACTTCTTCTT-3’	Integrated DNA Technologies	N/A
mCalcb.S: 5’-TGGAACAGGAGGAGCAAGAG-3’ AS: 5’-CACACCTCCTGATCTGCTCA-3’	Integrated DNA Technologies	N/A
mKiss1.S: 5’-AAGTGAAGCCTGGATCCACA-3’ AS:5’-TTAACGAGTTCCTGGGGTCC-3’	Integrated DNA Technologies	N/A
mPomc.S: 5’-ATGCCGAGATTCTGCTACAGT-3’ AS: 5’-TCCAGCGAGAGGTCGAGTTT-3’	Integrated DNA Technologies	N/A
mS14.S: 5’-TGGTGTCTGCCACATCTTTGCATC-3’ AS: AGTCACTCGGCAGATGGTTTCCTT	Integrated DNA Technologies	N/A
mMrgpra1	ThermoFisher	Assay ID: Mm01984314
mNpffr1	ThermoFisher	Assay ID: Mm01176033
mNpffr2	ThermoFisher	Assay ID: Mm00500040
mTacr1	ThermoFisher	Assay ID: Mm00436892
mIrf1	ThermoFisher	Assay ID: Mm01288580
mIrf2	ThermoFisher	Assay ID: Mm00515206
mCxcl10	ThermoFisher	Assay ID: Mm00445235
mIl12a	ThermoFisher	Assay ID: Mm00434169
mChil3	ThermoFisher	Assay ID: Mm00657889
mArg1	ThermoFisher	Assay ID: Mm00475988
Software and algorithms		
Biorender	Biorender	www.biorender.com
FlowJo v10.9.0	BD/Tree Star Inc.	https://www.flowjo.com/solutions/flowjo
GraphPad Prism v10	Prism	www.graphpad.com
ChEA3 - ChIP-X Enrichment Analysis Version 3	Keenan et al.^25^	https://maayanlab.cloud/chea3/
fastp v0.2	Chen et al.^77^	https://github.com/OpenGene/fastp
Salmon v1.1	Patro et al.^78^	https://github.com/COMBINE-lab/salmon
DEseq 2 v1.34	Love et al.^79^	https://bioconductor.org/packages/release/bioc/html/DESeq2.html
Seurat v4	Hao et al.^80^	https://satijalab.org/seurat/
Bioconda	Gruning et al.^81^	https://bioconda.github.io
Morpheus (heat map)	N/A	https://software.broadinstitute.org/morpheus
Harmony integration	Korsunsky et al.^82^	https://github.com/immunogenomics/harmony
scDbFfinder	Germain et al.^83^	https://bioconductor.org/packages/release/bioc/html/scDblFinder.html
Other		
BD LSRII cell analyzer	BD Biosciences	N/A
MoFlo XDP cell sorter	Beckman Coulter	N/A
MA900 cell sorter	Sony	N/A
ABI StepOnePlus	ThermoFisher	N/A

## Highlights:

Mrgpra1 is a neutrophil-expressed anti-inflammatory Mrgpr

Mrgpra1 drives anti-inflammatory neutrophils and inhibits activated neutrophils

A distinct population of neutrophils makes the Mrgpra1 ligand, neuropeptide FF (NPFF)

The Mrgpra1-NPFF axis inhibits IFNγ-driven anti-bacterial neutrophils
