# Supplementary material for: A GPCR-neuropeptide axis dampens hyperactive neutrophils by promoting an alternative-like polarization during bacterial infection
Source: Immunity. Author manuscript; Available in PMC 2025 Feb 13. (PMC10940224; doi:10.1016/j.immuni.2024.01.003)
Supplement: 1 [file NIHMS1960012-supplement-1.pdf]

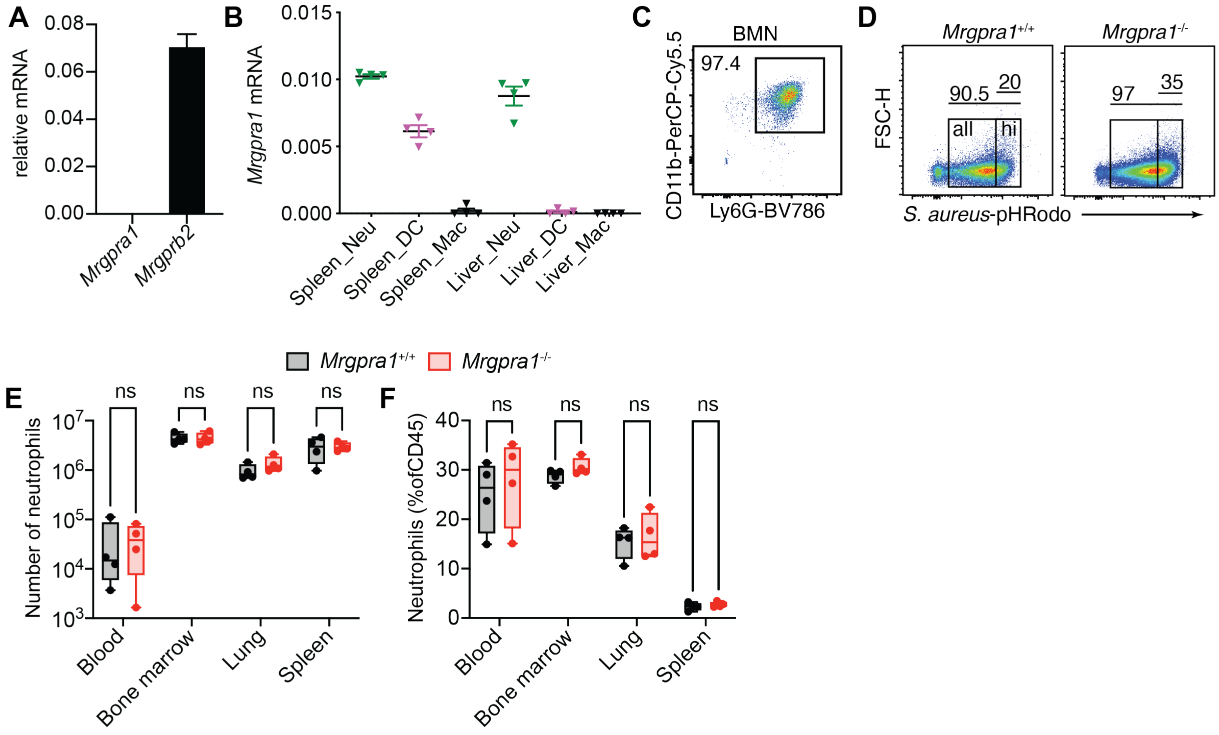

Figure S1. Mrgpra1 is a neutrophil gene that regulates activation. Related to Figure 1.
(A) *Mrgpra1* and *Mrgprb2* mRNA expression in cultured peritoneal mast cells from pooled mouse samples.
(B) *Mrgpra1* expression in sorted spleen and liver neutrophils (CD11b$^+$Ly6G$^+$), DCs (Ly6G$^-$F4/80$^-$CD11c^{hi}MHCII^{hi}) and macrophages (Ly6G$^-$F4/80$^+$), each dot is a technical replicate, representative of 2 independent sorts.
(C) Representative flow plot showing BMN purity of cells used in *in vitro* experiments. (D) Representative flow plot of *S. aureus*-pHRodo uptake by pulmonary neutrophils. (E) Numbers and (F) frequency of neutrophils in blood (100 uL), bone marrow (one femur), lung and spleen from naive *Mrgpra1*$^{+/+}$ and *Mrgpra1*$^{-/-}$ animals. Each dot (E, F) represents an animal (n=4). Male and female mice were used for all experiments (A-F) and data is representative of 2 experiments. Data are mean+SEM

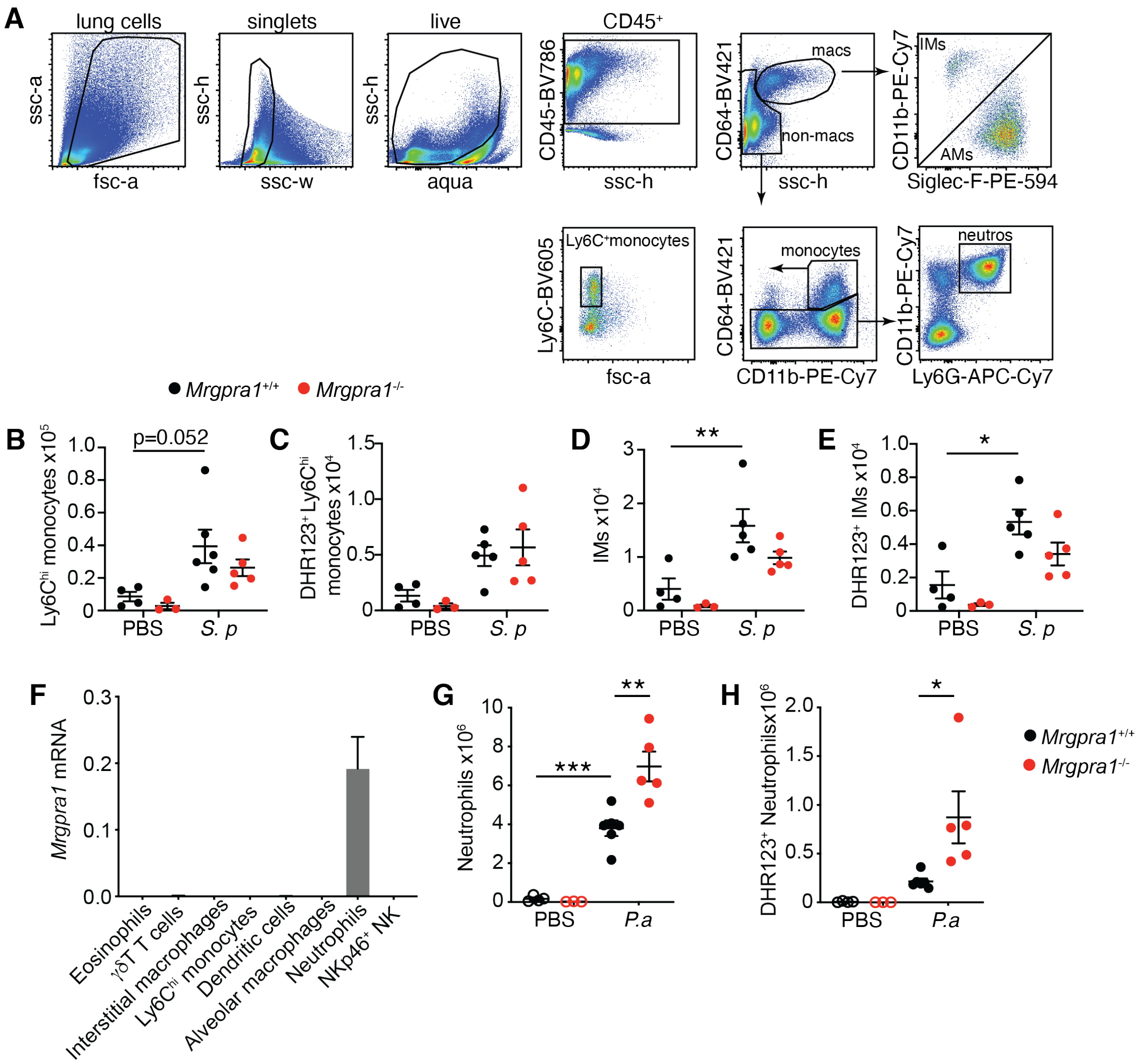

Figure S2. Mrgpra1 regulates the neutrophil, but not monocytes or macrophages, response to *S. pneumoniae* and *Pseudomonas aeruginosa.* Related to Figure 2. (A) Flow gating scheme to identify lung neutrophils, monocytes and macrophages. Mice were administered 2.5×10^5 S. pneumoniae (*S.p*) CFU i.t. and 24h later numbers of lung (B) $Ly6C^{hi}$ monocytes (C) $DHR123^+$ $Ly6C^{hi}$ monocytes, (D) IMs and (E) $DHR123^+$ IMs were enumerated. (F) Immune lung cells were sorted from *S. pneumoniae*-infected mice 24h after infection. Mice were administered 10^7 CFU of *P. aeruginosa* (*P.a*) i.t. and 24h later numbers of lung (G) neutrophils and (H) $DHR123^+$ neutrophils were enumerated. Each dot (B-E and G,H) represents an animal (n= 3-7). Male and female were used for experiments and data is representative of 2 experiments (a-h). $*p<0.05$, $**p<0.01$, $***p<0.001$.

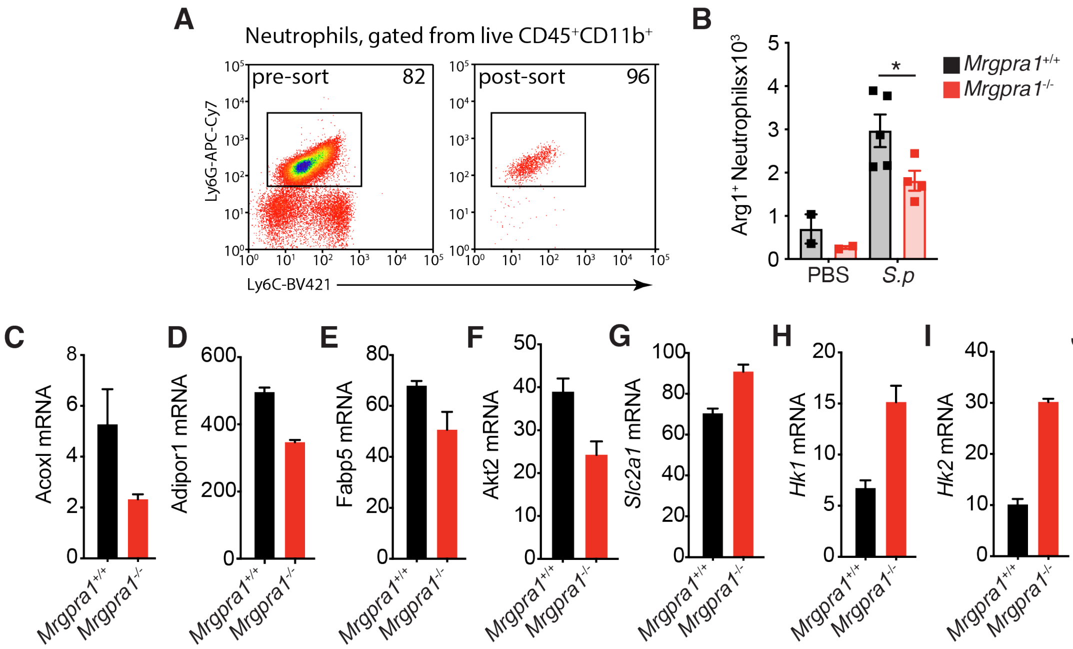

Figure S3. Mrgpra1 regulates alternative polarization and is associated with β-oxidation associated genes. Related to Figure 3. (A) Purity of flow sorted lung neutrophils from male mice. (B) Mice were administered 2.5x10^5 CFU of *S. pneumoniae* (*S.p*) and 24h later arginase-1$^+$ lung neutrophils were quantified, each dot is an animal (n=2-5). Levels of β-oxidation related genes (C) *Acoxl*, (D) *Adipor1*, (E) *Fabp5* and (F) *Akt2*, and glycolysis-associated genes (G) *Slc2a1*, (H) *Hk1*, (I) *Hk2*, and (J) *Pfkp* in lung neutrophils from *S. pneumonaie*-infected mice. Data is from our RNAseq dataset (C-J). Data is mean+sem and analyzed by one-way ANOVA. *p<0.05; **p<0.01; ***p<0.001.

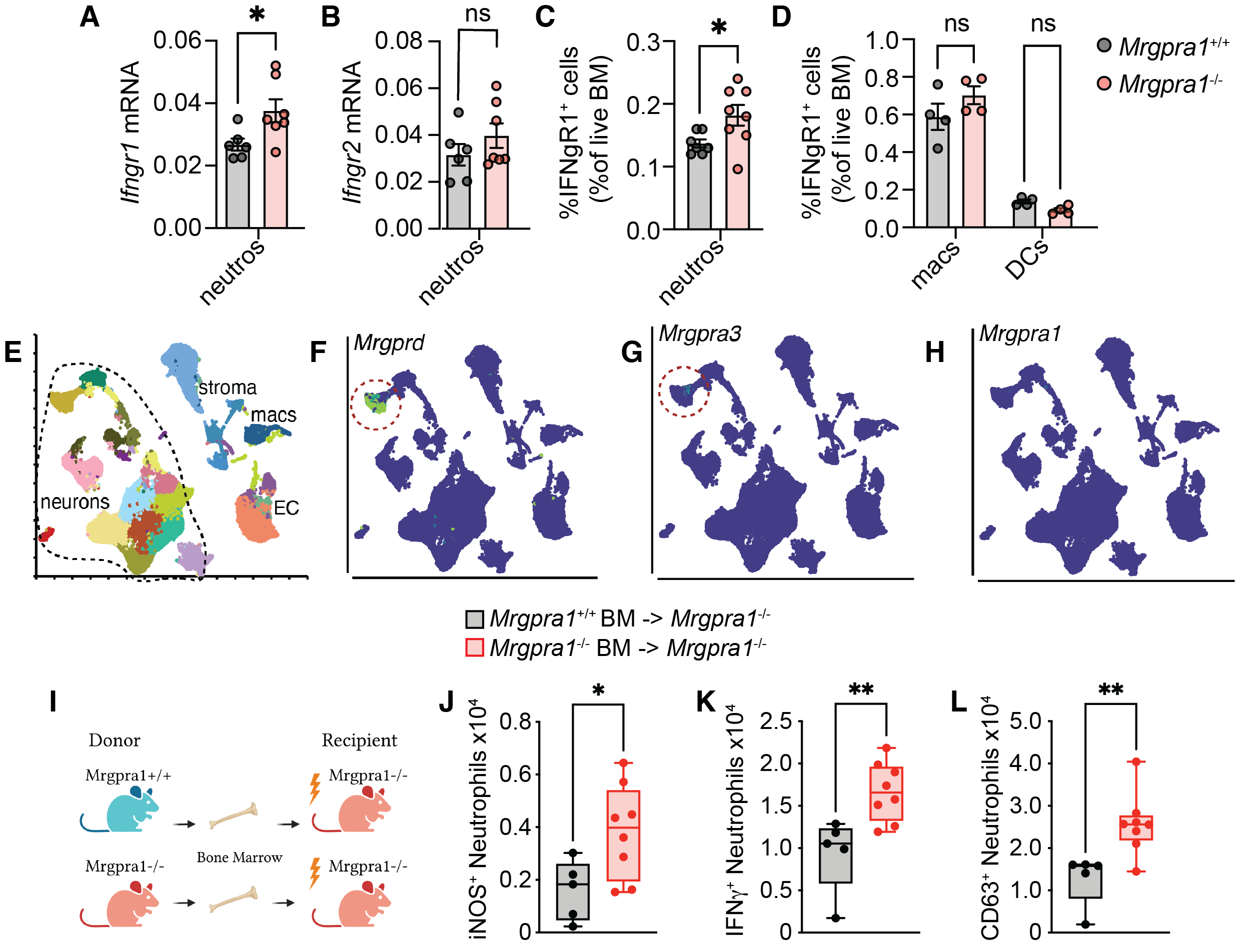

Figure S4. Mrgpra1 on hematopoeitic cells regulates neutrophil polarization. Related to Figure 4. Expression of (A) *Ifngr1* and (B) *Ifngr2* in BMNs (each dot is an individual replicate, pooled from 2 experiments). Expression of IFNgR1 on (C) neutrophils ($CD45^+CD11b^{hi}Ly6G^+$) (D) macrophages ($CD45^+CD64^+MerTK^+$) and DCs ($CD45^+CD64^-MerTK^-MHCII^{hi}CD11c^{hi}$) (each dot represents an animal, pooled or representative of 2 experiments). (E) UMAP (GSE192987) of scRNAseq data of mouse VG showing (F) *Mrgprd*, (G) *Mrgpra3* and (H) *Mrgpra1* expression. (I) Lethally-irradiated *Mrgpra1*$^{-/-}$ mice reconstituted with *Mrgpra1*$^{+/+}$ or *Mrgpra1*$^{-/-}$ bone marrow. Chimeric animals were treated with $2.5x10^5$ *S. pneumoniae* CFU and 24h later, BAL was harvested and analyzed for numbers of (J) $iNOS^+$, (K) $IFN\gamma^+$ and (L) $CD63^+$ neutrophils (each dot is an animal, n= 5-8). Data is mean+sem, analyzed by Student's T test and are pooled (A-C) or representative (D,J,K,L) of 2 experiments. Male and female mice were used. *$p<0.05$; **$p<0.01$.

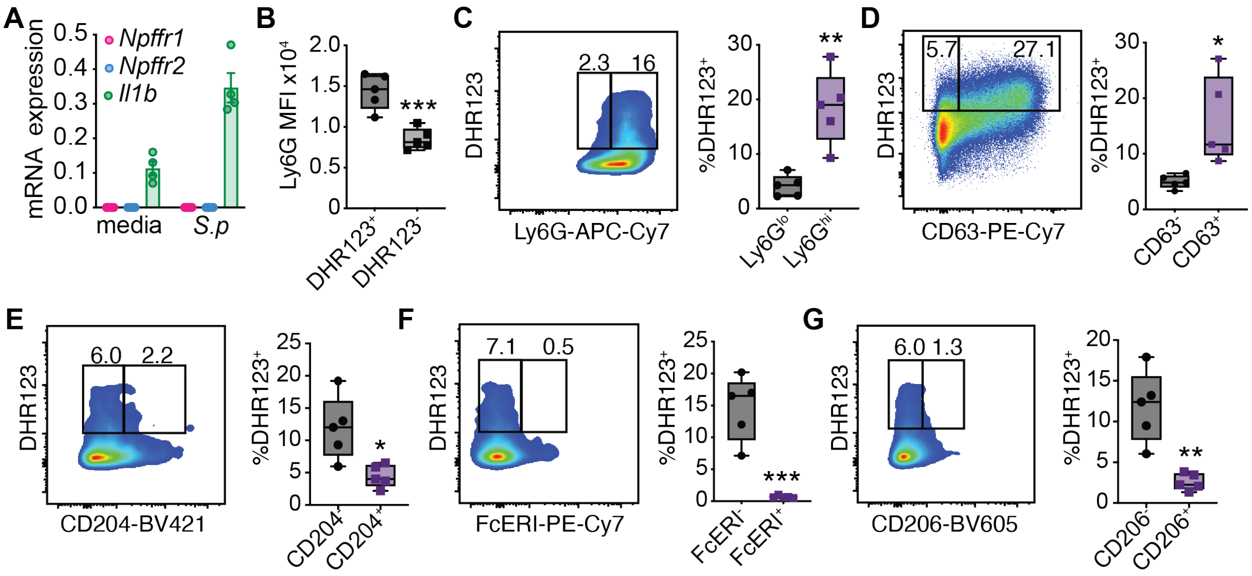

Figure S5. ROS⁺ neutrophils express higher markers of activation and less alternative markers. Related to Figure 5. (A) Expression of Npffr1, Npffr2 and Il1b in BMN treated with media or *S. pneumoniae* (MOI 1) for 4h (each dot is a technical replicate, representative of 2 independent experimens). Mice were exposed to *S. pneumoniae* i.t. and 24h later, BAL neutrophils were analyzed for (B) Ly6G MFI in DHR123$^+$ and DHR123$^-$ neutrophils, and for frequency of DHR123$^+$ neutrophils in (C) Ly6G^{lo} and Ly6G^{hi}, (D) CD63$^-$ and CD63$^+$, (E) CD204$^+$ and CD204$^-$, (F) FcER1$^+$ and FcER1$^-$, and (G) CD206$^+$ and CD206$^-$ fractions. Each dot (B-G) is an animal (n=5). Male and females were used in all experiments and data mean+SEM and is analyzed by Student's T test and representative of 2-3 independent experiments. *p<0.05; **p<0.01; ***p<0.001.

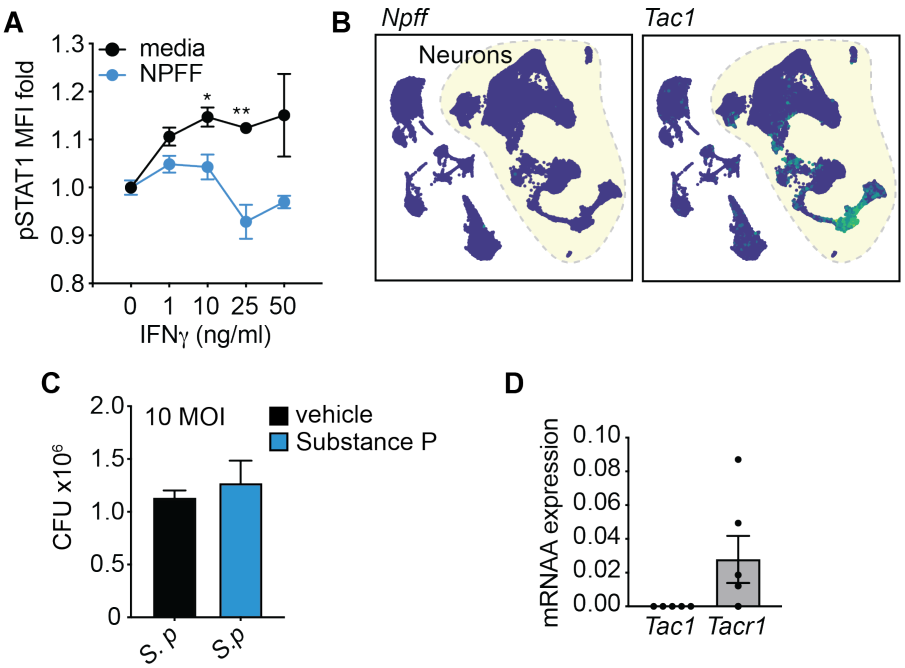

Figure S6. Substance P is expressed by VG neurons but does not inhibit neutrophils. Related to Figure 6
(A) phopsho-STAT1 in BMNs from wildtype mice pre-treated (4h) with NPFF before rIFNγ stimulation for 45 min, data is fold over media. (B) *Npff* and *Tac1* expression in mouse VG scRNAseq (GSE192987), highlighted in yellow are neurons. (C) remaining *S. pneumoniae* CFU (MOI 10) from BMNs pre-treated (4h) with media or substance P (25 uM). (D) *Tac1* and *Tacr1* mRNA expression in BMNs (each dot is a technical replicate). Male and female mice were tested (A,C and D) and data is representative (A, C) or pooled (D) from 2 independent expriments. Data is mean+sem and analyzed by T-test, *p<0.05**p<0.01.
